# Structural basis of ligand selectivity in FAD/NAD(P)H‐dependent dehydrogenases: insights from trypanothione reductase and type II NADH dehydrogenase

**DOI:** 10.1002/pro.70664

**Published:** 2026-06-20

**Authors:** Giulia Chiara Maria Perrone, Serena Spadone, Anna Lucia Francavilla, Sara Morselli, Sabino Todisco, Margherita Ortalli, Jairo Alfonso Mendoza‐Roldan, Valeria Scaglione, Maria Noemi Sgobba, Lorenzo Guerra, Federica Belluti, Stefania Varani, Domenico Otranto, Anna De Grassi, Mariateresa Volpicella, Ciro Leonardo Pierri

**Affiliations:** ^1^ Laboratory of Biochemistry, Structural and Molecular Biology, Department of Pharmacy—Pharmaceutical Sciences University of Bari “Aldo Moro” Bari Italy; ^2^ Department of Biosciences, Biotechnologies and Environment University of Bari “Aldo Moro” Bari Italy; ^3^ Section of Microbiology, Department of Medical and Surgical Sciences University of Bologna Bologna Italy; ^4^ Focalis Srl Mirandola MO Italy; ^5^ Microbiology Unit IRCCS Azienda Ospedaliero‐Universitaria di Bologna Bologna Italy; ^6^ Dipartimento di Medicina Veterinaria Università Degli Studi di Bari “Aldo Moro” Bari Italy; ^7^ Department of Pharmacy and Biotechnology University of Bologna Bologna Italy

**Keywords:** *Caldalkalibacillus thermarum*, docking selectivity analysis, drug repurposing, enzyme inhibition, FAD/NAD(P)H‐dependent dehydrogenases, homology modeling, *Leishmania infantum*, redox metabolism, structure‐based drug design, trypanothione reductase

## Abstract

FAD/NAD(P)H‐dependent dehydrogenases form a structurally conserved family of redox enzymes that participate in essential metabolic processes across parasites and higher organisms. Among them, trypanothione reductase (TR) is a key component of the redox metabolism of *Leishmania* species and represents an attractive target for antileishmanial drug development. However, because several flavoproteins share similar folds and cofactor‐binding architectures, the selectivity of TR inhibitors remains a critical issue during early drug discovery. To explore this aspect, we investigated the structural landscape of FAD/NAD(P)H‐dependent dehydrogenases in *Leishmania infantum* by integrating sequence analysis, structural modeling, docking simulations, and in vitro biochemical validation. Reciprocal sequence searches revealed 11 parasite flavoproteins structurally related to TR, including a dihydrolipoamide dehydrogenase (DLD)‐like protein, a type II NADH dehydrogenase (NDH2)‐like protein, and a dienoyl‐CoA reductase (deCoAR)‐like protein. Comparative docking analyses across parasite and mammalian homologs allowed us to examine potential cross‐reactivity patterns among these enzymes. Enzymatic assays performed on recombinant *L. infantum* TR (LiTR) and *Caldalkalibacillus thermarum* NDH2 (CtNDH2) confirmed that selected ligands can exert enzyme‐dependent effects. In particular, auranofin and, to a lesser extent, nitrofurazone inhibited LiTR, whereas a terpyridine–Pt‐derived compound strongly inhibited LiTR while stimulating CtNDH2 activity. Overall, the results illustrate how structurally related flavoproteins may accommodate common ligands while responding with distinct catalytic outcomes. The integrated computational and biochemical workflow presented here provides a practical framework for assessing ligand selectivity within the FAD/NAD(P)H‐dependent dehydrogenase family and may support the development of selective modulators targeting parasite redox metabolism.

## 1 INTRODUCTION

Leishmaniases are vector‐borne parasitic diseases caused by 53 *Leishmania* spp (Burza et al., 2018) of which approximately 20 are pathogenic to humans (Kaufer et al., 2017). These diseases affect millions of people worldwide, with a disproportionate burden on economically disadvantaged populations in tropical and subtropical regions. Human leishmaniases present in multiple clinical forms, such as cutaneous leishmaniasis (CL) and visceral leishmaniasis (VL), according to the localization and severity of the disease. Leishmania infantum is the most relevant zoonotic species, which has dogs as the main reservoirs and causes VL in many regions of South America, the Mediterranean basin, and West and Central Asia (Dantas‐Torres et al., 2012). The same species may also determine cutaneous and mucosal lesions, according to the individual immune response, being the most frequently diagnosed condition in the Mediterranean region (Serafim et al., 2020). Beyond the preventative measures available for limiting the infection in canine population and therefore the risk of human leishmaniosis, the mainstay treatments for leishmaniases in humans include pentavalent antimonials, amphotericin B, miltefosine, paromomycin and pentamidine (Zhang et al., 2025). Although the etiological agent is the same as that causing canine leishmaniosis, therapeutic protocols differ in order to minimize the risk of drug resistance and to preserve the efficacy of compounds that are essential for human treatments (Carrasco‐Martin et al., 2026; Krämer et al., 2025; Schäfer et al., 2024). Remarkably, for most of the mentioned compounds, the biological targets and mechanisms of action remain largely undefined (Gupta et al., 2023) and current therapeutic options for leishmaniasis are limited and present significant drawbacks, including toxicity, high costs, long‐course and invasive administration routes, and the emergence of drug‐resistant parasite strains. These issues are particularly critical in immunocompromised patients, who exhibit poor treatment outcomes and an increased susceptibility to drug toxicity (Burza et al., 2018). For these reasons, effective, safe, and short‐course oral therapies are urgently needed for both humans and dogs. In this context, a deeper understanding of parasite‐specific molecular targets and their modulation at the enzymatic level is essential to support the rational development and repurposing of antileishmanial agents.

Target‐based approaches to antileishmanial drug development primarily demand a detailed analysis and subsequent exploitation of biochemical differences between host and parasite biology (Gupta et al., 2023). Among the others, trypanothione reductase (TR) has emerged as a promising drug target due to its pivotal effect on the parasite's redox balance (Battista et al., 2020). Unlike mammals, which rely on glutathione reductase for maintaining intracellular redox homeostasis, *Leishmania* utilizes TR to reduce trypanothione disulfide into its active dithiol form. This fundamental difference in antioxidant metabolism makes LiTR an attractive target for selective drug design. At the same time, LiTR is among the best structurally characterized *Leishmania* enzymes, with multiple high‐resolution crystal structures available in complex with cofactors and inhibitors, making it particularly suitable for structure‐based investigations.

In this study, we explored LiTR structures complexed with inhibitors retrieved from the Protein Data Bank (PDB) to validate docking methodologies, define optimized gridboxes for structure‐based analyses through docking, and systematically investigate ligand–enzyme recognition within flavoprotein active sites.

However, despite its recognized value as a drug target, LiTR shares significant structural features with other members of the flavoprotein family, raising legitimate concerns regarding inhibitor selectivity and potential off‐target effects. To address this issue, we systematically examined FAD/NAD(P)H‐dependent dehydrogenases from *Leishmania infantum*, with particular attention to type II NADH dehydrogenase (NDH2), dihydrolipoamide dehydrogenase (DLD), and dienoyl‐CoA reductase, selected on the basis of their structural and functional relationships with LiTR (Trisolini et al., 2019). Structural superimposition and homology modeling were employed to compare the architecture of their cofactor‐ and inhibitor‐binding regions, enabling an estimation of relative binding affinities of TR‐directed ligands across these parasite enzymes. This comparative approach was designed to identify binding trends capable of distinguishing TR‐preferred compounds from ligands displaying broader affinity toward multiple flavoproteins. To provide experimental support for the computational predictions, selected molecules were further evaluated through in vitro enzymatic assays using recombinant LiTR and a reference NDH2 enzyme, thereby assessing enzyme‐dependent effects under controlled biochemical conditions. To extend the relevance of this framework beyond the parasite context, structurally related mammalian enzymes, including apoptosis‐inducing factor (AIF), glutathione reductase, thioredoxin reductase, and human dihydrolipoamide dehydrogenase, were also included in the comparative analysis (Dipol et al., 2025). These enzymes share conserved FAD/NAD(P)H‐dependent catalytic architectures and therefore represent plausible off‐targets in the development of LiTR inhibitors (Dipol et al., 2025). By integrating docking‐based comparisons across parasite and host homologs, we establish a structural basis for guiding the optimization of LiTR‐directed compounds with improved selectivity profiles. Although experimental validation in mammalian systems is beyond the scope of the present study, the proposed comparative framework may guide future efforts toward species‐ or enzyme‐selective flavoprotein modulators.

## 2 METHODS

### 2.1 Sampling of FAD/NAD(P)H dependent dehydrogenase sequences in *L. infantum* by blastp by using the sequences of crystallized FAD/NAD(P)H dependent dehydrogenases as query sequences

In order to identify possible flavoprotein candidates in *L. infantum*, a series of relevant protein sequences were used as queries to run several *blastp* analyses on the non‐redundant (nr) protein sequence database (https://www.ncbi.nlm.nih.gov/refseq/about/nonredundantproteins/) restricted to the taxon of the microorganism of interest (*L. infantum*, taxid:5671). The query sequences were selected according to previous results about FAD/NAD(P)H‐dependent dehydrogenases (Dipol et al., 2025; Trisolini et al., 2019), with specific reference to the human Apoptosis‐inducing factor (AIF, PDB ID 4bur; Ferreira et al., 2014), *Escherichia coli* NDH2 (PDB ID 4nwz; Heikal et al., 2014), human DLD (PDB ID 1zmd; Brautigam et al., 2005), *Aquifex aeolicus* sulfide:quinone oxidoreductase (PDB ID 3hyw; Marcia et al., 2009), *Marichromatiumgracile* glutathione disulfide reductase (GSR, PDB ID 2r9z; Van Petegem et al., 2007), LiTR (PDB ID 6i7n; Revuelto et al., 2019), *Plasmodium falciparum* thioredoxin reductase (TrxR, PDB ID 4j56; Fritz‐Wolf et al., 2013), *M. tuberculosis* disulfide reductase (PDB ID 1xdi; Argyrou et al., 2004), *Xanthobacter autotrophicus* NADPH‐dependent 2‐ketopropyl coenzyme M oxidoreductase/carboxylase (PDB ID 1mo9; Nocek et al., 2002), *Pseudomonas aeruginosa* mercuric reductase (PDB ID 4k7z; Lian et al., 2014), and *E. coli* dienoyl oxidoreductase (PDB ID 1ps9; Hubbard et al., 2003) (see Table 1). This set of query sequences is considered sufficiently representative to identify the majority of FAD/NAD(P)H‐dependent dehydrogenases in the *L. infantum* genome.

**TABLE 1:** Results of structure sampling and fold‐recognition analyses performed using pGenThreader and I‐TASSER on Leishmania infantum FAD/NAD(P)H‐dependent dehydrogenases.

Sequence annotation	PDB ID	Organism	pGenThreader annotation	Cofactor	Coverage	Identity	PDB ID	Organism	ITASSER annotation	Cofactor	Coverage	Identity
XP 001462998.1 TR	6awa	*P. putida*	DLD	FAD	0.95	0.27	1fea	*C. fasciculata*	TR	FAD	0.99	0.78
				AMP								
XP 001468025.1 DLD	5u8u	*P. aeruginosa*	DLD	FAD	0.97	0.49	2qae	*T. cruzi*	DLD	FAD	0.97	0.75
CAC9521120.1 AKGDH	5u8u	*P. aeruginosa*	DLD	FAD	0.89	0.23	6uzi	*E. anophelis*	DLD	FAD	0.9	0.25
XP 001467588.1 AKGDH	5u8u	*P. aeruginosa*	DLD	FAD	0.93	0.21	1dxl	*P. sativum*	DLD	FAD	0.93	0.24
XP 001466710.1 DLD	5u8u	*P. aeruginosa*	DLD	FAD	0.91	0.25	1dxl	*P. sativum*	DLD	FAD	0.91	0.25
XP 003392719.1 AoDH	5u8u	*P. aeruginosa*	DLD	FAD	0.84	0.24	1jeh	*S. cerevisiae*	DLD	FAD	0.86	0.25
XP 001469921.1 NDH2	5wed	*C. thermarum*	NDH2	FAD	0.72	0.29	4g6h	*S. cerevisiae*	NDH2	FAD	0.81	0.28
										NAI		
XP 003392329.1 NO	5n6g	*A. radiobacter*	NerA	FMN	0.96	0.41	1gwj	*P. putida*	Morphinone	FMN	0.99	0.41
									Reductase			
XP 003392330.1 nemiR	6myw	*G. oxidans*	ene‐reductase	FMN	0.43	0.39	1gwj	*P. putida*	Morphinone	FMN	0.41	0.2
									Reductase			
XP 001463176.1 deCoAR	6qkr	*E. coli*	naphtoyl‐CoA	FAD	0.87	0.26	1 ps9	*E. coli*	Dienoyl‐CoA	FAD	0.91	0.29
			Reductase	FMN					Reductase	FMN		
										NAP		
XP 001468165.1 deCoAR	6qkr	*E. coli*	naphtoyl‐CoA	FAD	0.87	0.28	1 ps9	*E. coli*	Dienoyl‐CoA	FAD	0.91	0.44
			Reductase	FMN					Reductase	FMN		
										NAP		
XP 001465165.1 G3PDH	2rgh	Streptococcus spp.	*α*‐GP oxidase	FAD	0.63	0.14	2rgo	Streptococcus spp	*α*‐GP oxidase	FAD	0.62	0.17

*Note*: For each predicted structural match, information on the corresponding crystallized proteins, including functional annotations and bound cofactors, was retrieved from the RCSB Protein Data Bank (PDB). For pGenThreader results, sequence coverage was calculated as the ratio between the alignment length and the full‐length query sequence, while percentage identity was derived directly from the pairwise alignment files. For I‐TASSER predictions, coverage corresponds to the Cov value and sequence identity to the Iden2 value reported in the “best threading templates” table for the highest‐ranked model.

Abbreviations: α‐GP, alpha‐glycerophosphate oxidase; AKGDH, α‐ketoglutarate dehydrogenase; AoDH, acetoin dehydrogenase; deCoAR, dienoyl‐CoA reductase; DLD, dihydrolipoamide dehydrogenase; G3PDH, glycerol‐3‐phosphate dehydrogenase; NDH2, type II NADH dehydrogenase; nemiR, N‐ethylmaleimide reductase; NO, NADH oxidase; TR, trypanothione reductase.

From this sampling round we obtained a group of *L. infantum* sequences, which we later used as query sequences in a reciprocal blastp analysis against the nr database restricted to the same taxon of *L. infantum* to ensure that all correlated sequences had been identified.

### 2.2 Searching for FAD/NAD(P)H dependent crystal dehydrogenases by using the sampled *L. infantum* FAD/NAD(P)H dependent dehydrogenase sequences as query sequences to run fold recognition tools

The obtained sequences, which lacked crystal structures, were investigated through fold recognition tools. pGenThreader (Lobley et al., 2009) and I‐TASSER (Zhang, 2008) were employed to search for distant homologous structures of our queried sequences. For each queried sequence, the crystal structures predicted as modeling templates with the highest confidence scores were selected for further investigation. In the case of pGenThreader, only templates classified as certain‐confidence hits were considered, whereas for I‐TASSER the top‐ranked threading templates associated with the highest confidence scores and sequence coverage were retained.

Furthermore, a keyword search of the available *L. infantum* flavoprotein sequences in the RCSB PDB (Berman et al., 2002) was performed to ensure that all crystal structures of interest had already been observed. Finally, the structures were superimposed and visually compared using molecular visualization tools such as PyMol (Delano & Bromberg, 2004), especially with the “*super*” command, then additional *blastp* analyses were launched on the sequences of a few selected crystal structures showing compelling structural relationships with one another.

### 2.3 Multiple sequence alignment of the sampled *L. infantum* FAD/NAD(P)H dependent dehydrogenase sequences and the most similar FAD/NAD(P)H dependent crystal structures

A multiple sequence alignment of our *L. infantum* FAD/NAD(P)H‐dependent dehydrogenase sequences and of the most similar FAD/NAD(P)H‐dependent crystal structures identified through fold recognition tools was performed in Jalview with its ClustalW implementation (v. 2.11.4.0) (Waterhouse et al., 2009).

Sequences were ordered according to their percentage of identical and similar residues, allowing the identification of clusters reflecting shared structural and functional features.

In addition, sequence similarity and sequence identity were quantified by using MatGat (v. 2.01) (Campanella et al., 2003). The results were then visually represented through R (v. 4.4.2) with the help of the *plot.matrix* and *viridis* packages (https://CRAN.R-project.org/package=Matrix).

The amino acid conservation of the investigated sequences was then explored and compared to known LiTR ligands. By using PyMol we highlighted residues located within 5 Å from our ligands of interest, namely the cofactors FAD, NAD(P)H, trypanothione, and two ubiquinone‐like molecules taken from type II NADH dehydrogenases of other species and introduced in LiTR by superimposition operations. These protein regions were explored under the assumption that, although not all the investigated enzymes are ubiquinone‐dependent, they may retain structurally accessible cavities related to UQ‐like binding regions, potentially conserved through divergent evolution and exploitable for structure‐based drug design. Finally, residues within 5 Å from the “high affinity ligands/inhibitors” observed in other crystallized TR structures retrieved from the RCSB PDB were investigated.

### 2.4 Homology modeling of relevant *L. infantum* flavoprotein sequences

Since no *L. infantum* FAD/NAD(P)H‐dependent dehydrogenases, except for TR, have been crystallized, we selected three *L. infantum* FAD/NAD(P)H dependent dehydrogenases apparently structurally/functionally related to NDH2, DLD, and dienoylCoA reductase, to build their homology models for subsequent comparative analysis and inhibitor‐selectivity assessment. With this aim, the sequences selected for modeling were aligned with the most similar crystal structure highlighted by using the above‐mentioned folding‐recognition tools. The resulting sequence‐structure pairwise alignments were then imported into SPDBV to generate the corresponding 3D homology models, according to previously described protocols (Giangregorio et al., 2022; Palmieri et al., 2023; Todisco et al., 2016). The alignments suggested by pGenThreader were employed for the modeling phase after being visually inspected and corrected to improve the alignment of known sequence motifs, ensuring the highest possible accuracy of the homology model. More in detail, based on the “sequence(to be modeled)/structure pairwise alignment” reproduced in SPDBV, all the lacking residues were added by using the Add Residue tool, and all the backbone breaks were ligated by using the Ligate Backbone tool. Afterwards, Swiss PDB Viewer's Energy Minimization was mainly utilized to solve clashes and/or physically unlikely bonds by conducting three cycles of 100 steps of steepest descent minimization. Clashes not solved by energy minimization were addressed using the Build Loop tool. After all the adjustments, the structures were energetically minimized until their free energy (∆G) converged to a value lower than −15,000 kJ/mol energy, and the correct 3D model packing was also verified by manual/visual inspection in PyMOL (Giangregorio et al., 2022; Palmieri et al., 2023; Todisco et al., 2016), before proceeding with the docking analyses.

### 2.5 Constitution of a library of known inhibitors of trypanothione reductase

Searching for inhibitors complexed with the sampled TR, the crystal structures suggested by pGenThreader and I‐TASSER were visually inspected. In addition, the RCSB PDB was investigated by using a keyword search for the presence of other TR crystal structures complexed with possible ligands of interest. The resulting set of small molecules, representing structurally diverse compounds crystallized in complex with LiTR and targeting distinct functional regions of the enzyme, was grouped based on a different set of spatially diverse targeted binding sites. The set of selected inhibitors used for re‐docking and docking simulations comprises the following seven molecules:

1. JV0, (4‐[[1‐(4‐Ethylphenyl)‐2‐Methyl‐5‐(4‐Methylsulfanylphenyl)pyrrol‐3‐Yl]methyl]thiomorpholine) crystallized in complex within LiTR in 4apn.pdb (Baiocco et al., 2013), consists of a 1,5‐diarylpyrrole‐based central core bearing a *para*‐ethyl‐phenyl and a *para*‐thiomethyl‐phenyl rings at N1 and C5 positions, respectively, along with a methyl at C2 and a thiomorpholinomethyl moiety at C3. The derivative inhibited LiTR, likely due to its capability to interact with the T(SH)2 binding site by a competitive mechanism of inhibition, thereby contacting residues involved in substrate binding, namely Glu18 and Tyr110 (Biava et al., 2011).
2. RDS, (6‐(sec‐butoxy)‐2‐((3‐chlorophenyl)thio)pyrimidin‐4‐amine or 6‐[(2R)‐butan‐2‐yl]oxy‐2‐(3‐chlorophenyl)sulfanylpyrimidin‐4‐amine), crystallized in complex within LiTR in 5ebk.pdb (Saccoliti et al., 2017), consists of a pyrimidine‐based diaryl sulfide derivative, also named RDS 777. It was originally identified as a reverse transcriptase inhibitor of human immunodeficiency virus type 1 (HIV‐1) (Costi et al., 2000). Further studies on this analogue evidenced the capability to inhibit *L. infantum* growth along with the inhibition of the TR by binding to the enzyme catalytic site and hydrogen bonding to some key residues, namely Glu466′, Cys57 and Cys52 (Saccoliti et al., 2017).
3. MWT (1‐[2‐[5‐[4‐(4‐aminobutyl)‐3‐methyltriazol‐1‐ium‐1‐yl]‐2‐[4‐(2‐phenylethyl)‐1,3‐thiazol‐2‐yl]phenoxy]ethyl]imidazolidin‐2‐one) crystallized in complex with LiTR in 6t95.pdb (de Lucio et al., 2022). The homodimeric nature of LiTR (Baiocco et al., 2013) suggested that disrupting the α‐helix‐mediated protein–protein interactions (PPIs), responsible for enzyme dimerization, could represent a successful inhibition strategy. In this context, the peptide PKIIQSVGIS‐Nle‐K‐Nle showed a remarkable effect against enzyme dimerization (Toro et al., 2013). Thus, by applying an α‐helical mimetic approach, MWT, a 1,2,3‐triazolium salt‐based analogue was rationally designed. The compound inhibited LiTR with low‐μM potency, displaying a competitive inhibition mechanism of action; moreover, it acted as a dimerization disruptor.
4. H6H (N‐(4‐aminobutyl)‐N‐(2‐amino‐2‐oxoethyl)‐7‐(3‐amino‐3‐oxopropyl)‐4‐(dimethylamino)‐2‐(2‐naphthalen‐2‐ylethylamino)pyrrolo[2,3‐d]pyrimidine‐6‐carboxamide) crystallized in complex with LiTR in 6i7n.pdb (Revuelto et al., 2019). H6H was rationally designed by purposely functionalizing the pyrrolo[2,3‐d]pyrimidine heterocycle, selected for its conformational rigidity, into specific positions. The selected functions aimed to mimic the key residues, namely lysine (K), glutamine (Q), and isoleucine (I), of the lead peptide‐based analogue PKIIQSVGIS‐Nle‐K‐Nle, regarded as a proteomimetic agent, that is, able to disrupt α‐helix‐mediated PPI, thus affecting LiTR dimerization. In particular, position 2 was decorated with a hydrophobic naphthalene‐ethylamino moiety, while an aminobutyl group was introduced in the carboxamide moiety in position 6, and a 3‐amino‐3‐oxopropyl moiety side chain was inserted in the N‐7. The derivative H6H proved to moderately inhibit *Li*TR by binding to the crucial cys52 and cys57 residues (Revuelto et al., 2019).
5. TS8 (2,3,4,6‐tetra‐O‐acetyl‐1‐thio‐β‐D‐glucopyranose, also known as auranofin, an approved drug for arthritis treatment, https://go.drugbank.com/drugs/DB00995) was crystallized in complex with LiTR in 2yau.pdb (Ilari et al., 2012) (and also with the thioredoxin reductase from the nematoda *Brugia malayi* in 7put.pdb; Fata et al., 2022). It is structurally composed of an acetylated β‐D‐thioglycosyl (acetyl‐β‐glycoside) moiety that is prone to undergo Michael addition reaction (Witczak et al., 2007) to electron‐rich substrates such as α,β‐unsaturated carbonyl compounds pharmacophores. It is largely exploited as a synthon for synthesizing thio‐glycoconjugates, regarded as promising therapeutic candidates (Dere et al., 2011; Pachamuthu & Schmidt, 2006).
6. WPF (3‐(6‐chloro‐2‐methyl‐4‐p‐tolylquinazolin‐3(4H)‐yl)‐N,N‐dimethylpropan‐1‐amine) crystallized in complex with *Trypanosoma brucei* TR (TbTR) in 2wpf.pdb (Patterson et al., 2011). consisting of a 3,4‐dihydroquinazoline core, showing to potently affect *T. brucei* growth (EC_50_ = 0.73 μM), likely due to effective inhibition of TbTR enzyme. A group of WPF related ligands show a Ki toward *Tb*TR in the low micromolar range (0.8–9 μM; Patterson et al., 2011).
7. BVN (2‐(diethylamino)ethyl 4‐((3‐(4‐nitrophenyl)‐3‐oxopropyl)amino)benzoate) crystallized in complex with LiTR in 6er5.pdb (Turcano et al., 2018), consisting of an analogue of 3‐amino‐1‐arylpropan‐1‐one scaffold, owing to its strong potency against LiTR (IC_50_ value of 7.5 μM) alongside a remarkable selectivity for human glutathione reductase, has emerged from a high‐throughput screening campaign (Colotti et al., 2020).

### 2.6 Redocking of the sampled inhibitors on the sampled crystal structures

To validate the accuracy of the docking protocol, redocking simulations of FAD, NADPH, trypanothione, and ubiquinone‐like cofactors, together with the identified inhibitors, were performed on LiTR (4apn.pdb, chain B). In addition, redocking of FAD and NADH was carried out on selected human flavoproteins, including dihydrolipoamide dehydrogenase (1zmd.pdb, chain C, in the oxidized disulfide state involving Cys45 and Cys49), apoptosis‐inducing factor (AIF, 4bur.pdb, chain A), glutathione reductase (1grb.pdb, chain A), and thioredoxin reductase (TrxR, 2j3n.pdb, chain D), representing relevant potential off‐targets of LiTR inhibitors in *H. sapiens*.

The redocking analysis was performed on monomeric forms obtained from the RCSB PDB. Each PDB file was inspected and prepared prior to redocking. Concerning redocking on the LiTR crystal structure (4apn.pdb), the analysis was performed with AutoDock4 (v. 4.2.6) and MGLTools (v. 1.5.7) (Morris et al., 2009) using default settings (150 populations, 2,500,000 evaluations and 27,000 generations). All gridboxes have a spacing of 0.281 Å. When ligands failed to produce satisfactory docking scores or reliable poses on the monomer, the dimeric form was additionally evaluated, but only in cases where crystallographic data or preliminary docking results indicated potential interactions at the monomer–monomer interface. In these instances, docking on the dimer was used to provide additional structural context and improve pose stability. Nevertheless, for all ligands and targets, docking simulations were systematically repeated and evaluated on the monomeric form, which was retained as the primary framework for comparative analyses. Trypanothione was docked in both reduced and oxidized forms, as the reduced molecule adopts an extended conformation that may influence pose reconstruction. In contrast, glutathione was analyzed only in its oxidized form (GSSG), since crystallographic evidence indicates that GSSG (taken from 1gra.pdb (Karplus & Schulz, 1989)) overlaps with the two reduced GSH molecules (seen in 1gre.pdb (Karplus & Schulz, 1989)) in related structures and provides a stable representation of the catalytic binding mode. Preliminary docking confirmed that GSSG yielded consistent poses, and therefore reduced glutathione was not further considered.

The gridboxes that better reproduced LiTR‐ligand crystallized complexes (in terms of RMSD between the docked complexes and the crystallized complexes) were used as starting gridboxes for running all the performed docking simulations aiming to investigate the selectivity of the investigated ligands for LiTR and the other *L. infantum* FAD/NAD(P)H‐dependent dehydrogenases modeled by SPDBV, after having superimposed the generated 3D models on the crystallized LiTR.

Where the resulting root mean square deviation of atomic coordinates (refRMS) calculated between docked and reference ligand poses after structural superimposition was above 3 Å, it was attempted to lower the refRMS value by redocking the ligand on the dimeric LiTR to provide the algorithm with additional structural context and improve the docking pose. In order to benchmark the obtained results, experimental inhibition constants were retrieved from the literature (Angiulli et al., 2015) and the BRENDA database (Schomburg et al., 2002) to provide an experimental reference for comparison.

### 2.7 Docking of high‐affinity ligands, crystallized within LiTR, on the generated homology models of other *L. infantum* FAD/NAD(P)H dependent dehydrogenases structurally related to LiTR

Each homology model was subjected to the same docking protocol established during the redocking phase. In this case, the ligand/cofactor gridboxes were established starting from the gridboxes used for specific cofactors/ligands in LiTR, after the superimposition of the generated 3D models with LiTR. Inhibitor gridboxes were kept unchanged; minor adjustments to cofactor gridboxes were performed based on small deviations of atomic coordinates of FAD, NAD(P)H, UQ in the crystallized proteins used for modeling *L. infantum* sequences.

In detail, the XP 001469921.1 model was built based on the *E. coli* type II NADH dehydrogenase (4g6h.pdb; Feng et al., 2012), whereas the XP 001468165.1 model and the XP 001468025.1 model were built based on the *E. coli* 2,4‐dienoyl‐CoA reductase (deCoAR, 1ps9.pdb; Hubbard et al., 2003) and on the human dihydrolipoamide dehydrogenase (1zmd.pdb) structures, respectively. In each case, the FAD and the NAD(P)H gridboxes were modified based on these cofactors' positions in the corresponding type II NADH dehydrogenase (4g6h.pdb); dihydrolipoamide dehydrogenase (1zmd.pdb; Brautigam et al., 2005), and 2,4‐dienoyl‐CoA reductase (1ps9.pdb; Hubbard et al., 2003).

When enzyme‐specific structural features of the generated models overlapped with the binding site of specific LiTR inhibitors, docking of those inhibitors was not performed to avoid biologically implausible poses. As before, we searched the literature for in vitro *𝐾*_*i*_ values of the redocked inhibitors for comparative purposes (Baiocco et al., 2013; de Lucio et al., 2022; Ilari et al., 2012; Patterson et al., 2011; Revuelto et al., 2019; Saccoliti et al., 2017; Turcano et al., 2018).

### 2.8 Selectivity analysis by docking of known FAD/NAD(P)H‐dependent dehydrogenase inhibitors on LiTR and CtNDH2

**FIGURE 1:**
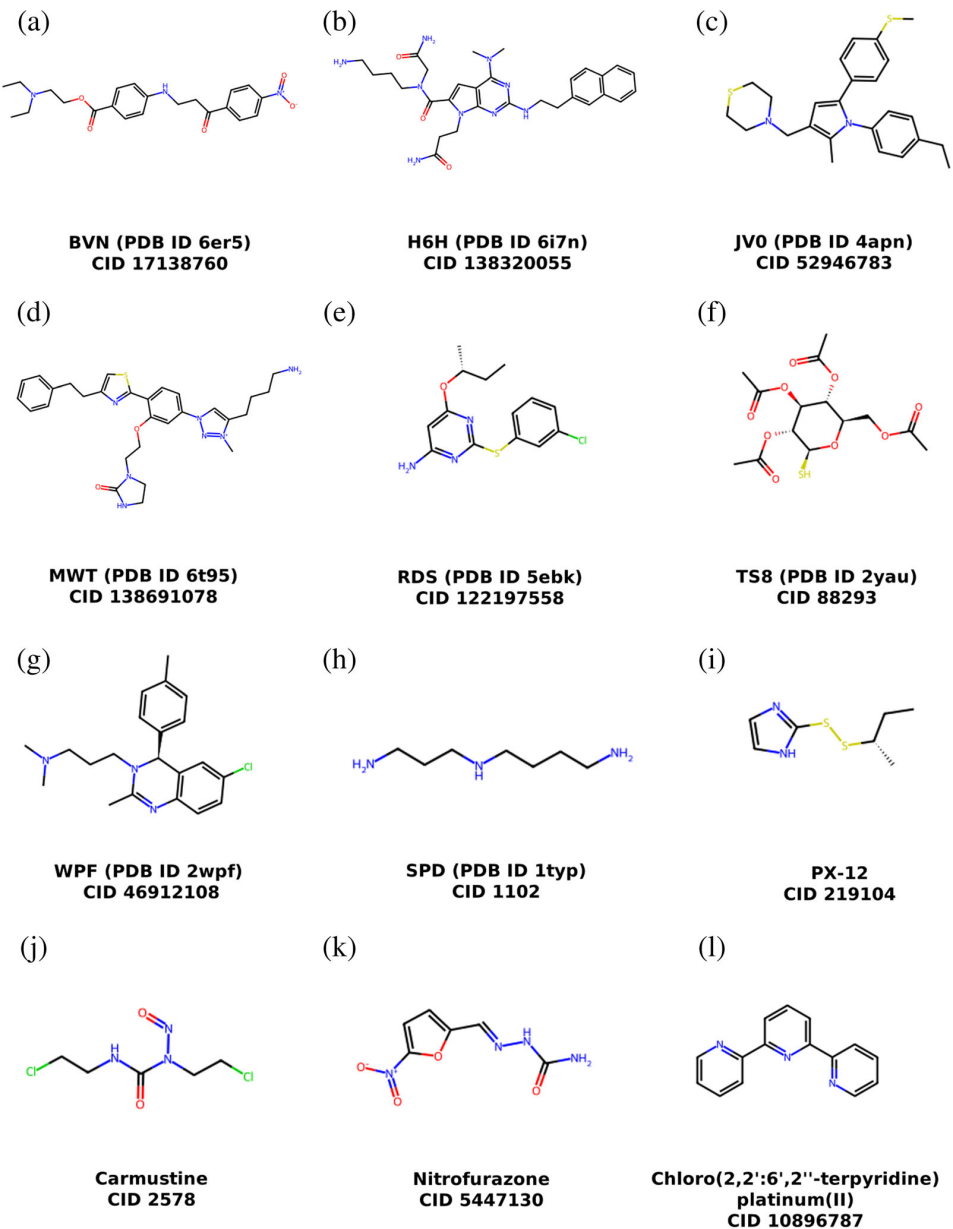
2D structures of the investigated inhibitors, as obtained from the RCSB PDB. For each compound, its PDB Chemcal ID is reported (where available), then the PDB ID of the crystal from which the co‐crystallized pose of interest was extracted, and finally the PubChem CID. Starting from the first row, the compounds depicted are BVN (2‐(Diethylamino)ethyl 4‐((3‐(4‐nitrophenyl)‐3‐oxopropyl)amino)benzoate) in panel (a), H6H (N‐(4‐aminobutyl)‐N‐(2‐amino‐2‐oxoethyl)‐7‐(3‐amino‐3‐oxopropyl)‐4‐(dimethylamino)‐2‐(2‐naphthalen‐2‐ylethylamino)pyrrolo[2,3‐d]pyrimidine‐6‐carboxamide) in panel (b) and JV0 (4‐[[1‐(4‐Ethylphenyl)‐2‐Methyl‐5‐(4‐Methylsulfanylphenyl)pyrrol‐3‐Yl]methyl]thiomorpholine) in panel (c). Then, in the second row the compounds depicted are MWT (1‐[2‐[5‐[4‐(4‐Aminobutyl)‐3‐methyltriazol‐1‐ium‐1‐yl]‐2‐[4‐(2‐phenylethyl)‐1,3‐thiazol‐2‐yl]phenoxy]ethyl]imidazolidin‐2‐one) in panel (d), RDS (6‐(sec‐butoxy)‐2‐((3‐chlorophenyl)thio)pyrimidin‐4‐amine 6‐[(2R)‐butan‐2‐yl]oxy‐2‐(3‐chlorophenyl)sulfanylpyrimidin‐4‐amine) in panel (e) and TS8 (2,3,4,6‐Tetra‐O‐acetyl‐1‐thio‐β‐D‐glucopyranose) in panel (f). In the third row, the compounds depicted are WPF (3‐(6‐Chloro‐2‐methyl‐4‐p‐tolylquinazolin‐3(4H)‐yl)‐N,N‐dimethylpropan‐1‐amine) in panel (g), SPD (N′‐(3‐aminopropyl)butane‐1,4‐diamine) in panel (h) and PX‐12 (2‐(butan‐2‐yldisulfanyl)‐1H‐imidazole) in panel (i). Lastly, in the bottom row the compounds depicted are carmustine (1,3‐bis(2‐chloroethyl)‐1‐nitrosourea) in panel (j), nitrofurazone ([(E)‐(5‐nitrofuran‐2‐yl)methylideneamino]urea) in panel (k) and chloro(2,2′:6′2″‐terpyridine)platinum(II) (chloroplatinum(1+);2,6‐dipyridin‐2‐ylpyridine) in panel (l).

To explore structural relationships between the known FAD/NAD(P)H‐dependent dehydrogenases and to perform a preliminary selectivity analysis, docking simulations were carried out with characterized FAD/NAD(P)H‐dependent dehydrogenase inhibitors (Figure 1) on both the LiTR (PDB ID 4apn) and the CtNDH2 (PDB ID 5wed).

Five compounds were chosen:

- Chloro(2,2′:6′,2″‐terpyridine)platinum, a derivative of the terpyridine platinum (TPT) class of inhibitors of human thioredoxin reductase, targeting an allosteric site of the protein (PDB ID 2zzb; Lo et al., 2009);
- Carmustine, a GSR inhibitor hypothesized to be able to bind the NADPH site of the mentioned enzyme (Begum et al., 2021);
- PX‐12, which is capable of reversibly covalently binding human thioredoxin, and non‐competitively inhibiting human thioredoxin reductase (Kirkpatrick et al., 1998; Oblong et al., 1994; Ramanathan et al., 2007). Its binding site is not known;
- Nitrofurazone, from which recently new derivatives were found to be able to inhibit TR, supposedly binding the trypanothione site (Andrés‐Rodríguez et al., 2025);
- Spermidine (SPD), a precursor of trypanothione, is known to interact with several systems and has been co‐crystallized with *C. fasciculata* TR (PDB ID 1typ; Bailey et al., 1993) as a component of oxidized trypanothione.

Each compound was docked in its experimentally known or hypothesized binding site on TR, using the same gridboxes as previously established for each site, aside from chloro(2,2′:6′,2″‐terpyridine)platinum, whose site had not been previously explored.

In this case, the gridbox was centered on coordinates (–42.222, 36.98, –21.366), using 41 × 19 × 25 gridpoints and spacing of 0.281 Å, based on comparative structural analysis and superimposition of LiTR and thioredoxin reductase.

PX‐12 was not docked due to the lack of reliable structural information regarding its binding site; however, it was tested in the in vitro assays described below.

### 2.9 Expression and purification of LiTR and CtNDH2

The coding sequences LiTR (XP_001462998.1) and CtNDH2 (WP_007502350.1) were cloned into the pET‐21b(+) expression vector. For affinity purification, a 6×His tag was fused to the N‐terminus of LiTR and to the C‐terminus of CtNDH2; in both constructs, a thrombin cleavage site was included upstream of the affinity tag.

The resulting plasmids were transformed into *E. coli* BL21(DE3) for LiTR expression and into *E. coli* C41(DE3) for CtNDH2 expression. Transformed cells were grown at 37°C in LB medium supplemented with 0.1 mg/mL ampicillin until an optical density at 600 nm (OD_600_) of 0.4–0.8 was reached. Protein expression was induced by the addition of isopropyl β‐D‐1‐thiogalactopyranoside (IPTG) at a final concentration of 0.7 mM for 20 h for *Li*TR and 1 mM for 4 h for CtNDH2, as previously described (Heikal et al., 2014).

Following induction, cells were harvested by centrifugation at 6000 rpm (3.945 × g) for 10 min at 4°C, resuspended in lysis buffer (50 mM NaH_2_PO_4_, 300 mM NaCl, 10 mM imidazole, pH 8.0), and disrupted using a French press. Cell lysates were centrifuged at 13,000 rpm (18,516 × g) for 25 min at 4°C to isolate the soluble fraction. All centrifugation steps were performed using a Fiberlite F15‐6 × 100y fixed‐angle rotor (Thermo Scientific). The supernatant was subsequently incubated overnight at 4°C with Ni‐NTA Agarose resin (QIAGEN, Hilden, Germany) under constant agitation.

Proteins were purified by affinity chromatography using a stepwise imidazole gradient (10, 50, 100, 150, and 300 mM). *Li*TR was eluted at imidazole concentrations between 10 and 100 mM, whereas CtNDH2 was eluted at 50 and 100 mM. Eluted fractions were concentrated and buffer‐exchanged using Amicon Ultra centrifugal filters (3 kDa molecular‐weight cutoff; Merck Millipore, Burlington, USA). *Li*TR was stored in 40 mM HEPES (pH 7.5), 1 mM EDTA, and 10% (v/v) glycerol, while CtNDH2 was stored in 50 mM Tris‐HCl (pH 8.0), 150 mM NaCl, and 10% (v/v) glycerol. Final protein concentrations were estimated by SDS‐PAGE using a bovine serum albumin (BSA) standard curve ranging from 1 to 5 μg.

### 2.10 Activity assays on recombinant LiTR and CtNDH2

The enzymatic activities of LiTR and CtNDH2 were determined spectrophotometrically by monitoring the oxidation of NADPH and NADH, respectively, at 340 nm using a Varian Cary® 50 UV‐Vis Spectrophotometer (Agilent, Santa Clara, USA). All assays were carried out in a final volume of 1 mL in 1 cm path length quartz cuvettes. Reaction mixtures contained the appropriate enzyme, its specific substrate (trypanothione for LiTR and decylubiquinone for CtNDH2), and saturating concentrations of either NADPH or NADH, respectively.

LiTR activity was measured by incubating the enzyme at a final concentration of 10 nM in the reaction buffer (40 mM HEPES, pH 7.5, 1 mM EDTA) with trypanothione for 2 min; the reaction was then initiated by the addition of NADPH. To determine the Vmax and apparent Km for trypanothione, enzymatic activity was measured across a range of substrate concentrations (30, 50, 100, 150, 250, 350, 450 μM) while maintaining NADPH at a constant concentration of 100 μM (Rodrigues et al., 2012).

CtNDH2 activity was assayed by incubating the enzyme at a final concentration of 20 nM in the reaction buffer (50 mM Tris‐HCl, pH 8, 150 mM NaCl) with decylubiquinone (dissolved in ethanol) for 2 min. The reaction was initiated by adding NADH. To determine the Vmax and apparent Km for decylubiquinone, activity was measured by varying the substrate concentration (25, 50, 75, 110, 150, 200, 250, 350, 450 μM) while keeping NADH at a final concentration of 200 μM (Heikal et al., 2014).

Specific enzymatic activity was calculated from the initial linear slopes of absorbance decrease at 340 nm, using the molar extinction coefficient for NADH/NADPH (ε340 = 6220 M^−1^ cm^−1^) and expressed as μmol × min^−1^ × mg protein^−1^.

Kinetic parameters (Vmax and Km) were derived by non‐linear regression analysis of the specific activities plotted against substrate concentrations, using GraphPad Prism version 10.5.0 (774) for Windows (GraphPad Software, Boston, Massachusetts, USA).

### 2.11 Activity assays in the presence of the investigated molecules

The enzymatic activities of LiTR and CtNDH2 were assessed in the presence of a panel of small molecules prepared as follows: (i) auranofin (1 mM, diluted from a 50 mM stock solution in DMSO); (ii) chloro(2,2′:6′,2″‐terpyridine)platinum (10 mM); (iii) spermidine (20 mM); (iv) PX‐12 (20 mM, dissolved in ethanol); (v) nitrofurazone (2 mM, diluted from a 100 mM stock in DMSO); and (vi) 20 carmustine (20 mM). All aqueous solutions were prepared using ultrapure water.

All assays were conducted in a final volume of 1 mL using 1 cm path‐length cuvettes, under the same experimental conditions described above. Each compound was tested at a final concentration of 100 μM. In the case of CtNDH2, nitrofurazone was assayed at a lower concentration (50 μM) to minimize background absorbance arising from the combined presence of nitrofurazone and decylubiquinone, both of which exhibit significant absorbance in the UV–visible range. The enzymes were used at the concentrations described in Section 2.10 and were pre‐incubated for 2 min with 50 μM of their respective substrates in the presence of the test compound. Reactions were subsequently initiated by the addition of the appropriate reducing cofactor. Negative control reactions contained the complete reaction mixture in the absence of inhibitors. To exclude solvent‐related effects on enzymatic activities, parallel control assays were performed using ultrapure water. Additional control assays for auranofin and PX‐12 included DMSO (0.2% v/v) and ethanol (0.5% v/v), respectively, at concentrations matching those used in the corresponding test samples.

## 3 RESULTS

### 3.1 Flavoprotein homologous sequences in *L. infantum*

The *blastp* of a selected set of crystallized FAD/NAD(P)H‐dependent dehydrogenases (Dipol et al., 2025; Trisolini et al., 2019) from different organisms (Table 1) against the *L. infantum* taxon yielded 12 protein sequences (Table 2), as confirmed by reciprocal *blastp* restricted on the same taxon. The retrieved sequences were annotated based on their similarity with other previously characterized enzymes (Table 1).

**TABLE 2:** Cofactors and ligands identified from the crystal structures of Leishmania infantum trypanothione reductase (TR) and structurally related flavoproteins and selected for redocking and docking analyses.

PDB_ID	Annotation	Crystallized cofactors substrates, other ligands	Organism	Blastp best hit/NCBI annotation
4bur	AIF	*FAD*, *NAD* ^+^, *SO*_4_^2−^	*Homo sapiens*	NP_665811.1 AIF
4nwz	NDH2	*FAD*	*E. coli*	WP_007502350.1
1zmd	DLD	*FAD*, *NAI*, *SO*_4_^2−^	*H. sapiens*	XP 001468025.1 DLD
				XP 001462998.1 TR
				XP 001466710.1 DLD
				CAC9521120.1 AKGDH
				XP 003392719.1 AoDH
3hyw	Sulfide:quinone oxidoreductase	*FAD*, *DCQ*, *LMT*, *PS9*, *SO*_4_^2−^, *H*_2_ *S*	*A. aeolicus*	WP_010881436.1 NDH2
2r9z	Glutathione reductase	*FAD*, *Ni* ^2+^, *Cl* ^−^	*M. gracile*	XP 001462998.1 TR
				XP 001468025.1 DLD
				XP 003392719.1 AoDH
				XP 001465165.1 G3PDH
6i7n	Trypanothione reductase	*FAD*, *H6H*, *SO*_4_^2−^, *GOL*	*L. infantum*	XP 001462998.1 TR
				XP 001468025.1 DLD
4j56	Thioredoxin reductase	*FAD*, *Thioredoxin*	*P. falciparum*	XP 001462998.1 TR
				XP 001468025.1 DLD
				CAC9521120.1 AKGDH
1xdi	Disulfide reductase	*FAD*	*M. tubercolosis*	XP 001466710.1 DLD
				XP 001462998.1 TR
1mo9	NADPH dependent 2‐ketopropyl coenzyme M oxidoreductase/carboxylase	*FAD*, *KPC*	*X. autotrophicus*	XP 001468025.1 DLD
				XP 001462998.1 TR
4k7z	Mercuric reductase	*FAD*, *NAP*, *Hg* ^2+^, *SO*_4_^2−^, *GOL*	*P. aeruginosa*	XP 001468025.1 DLD
				XP 001466710.1 DLD
				XP 003392719.1 AoDH
				CAC9521120.1 AKGDH
				XP 001469921.1 NDH2
1ps9	deCoAR	*MDE*, *FAD*, *NAP*, *FMN*, *SF*4, *Cl* ^−^	*E. coli*	XP 001468165.1 deCoAR
				XP 001463176.1 deCoAR
				XP 003392330.1 nemiR
				XP 003392329.1 NO

*Note*: Reported Km values were retrieved from the literature (Schäfer et al., 2024 and references therein), where available. The table summarizes the Protein Data Bank (PDB) entries, associated cofactors or bound ligands, and the corresponding target enzymes.

Abbreviations: AIF, apoptosis‐inducing factor; NDH2, type II NADH dehydrogenase; DLD, dihydrolipoamide dehydrogenase; TR, trypanothione reductase; deCoAR, dienoyl‐CoA reductase; AKGDH, α‐ketoglutarate dehydrogenase; AoDH, acetoin dehydrogenase; NAI, 1,4‐dihydronicotinamide adenine dinucleotide; NAP, NADP^+^ (nicotinamide adenine dinucleotide phosphate); LMT, dodecyl‐β‐D‐maltoside; PS9, octathiocane; H6H, N‐(4‐aminobutyl)‐N‐(2‐amino‐2‐oxoethyl)‐7‐(3‐amino‐3‐oxopropyl)‐4‐(dimethylamino)‐2‐(2‐naphthalen‐2‐ylethylamino)pyrrolo[2,3‐d]pyrimidine‐6‐carboxamide; KPC, 2‐(2‐ketopropylthio)ethanesulfonate; GOL, glycerol; MDE, 5‐mercaptoethanol‐2‐decenoyl‐coenzyme A; SF4, [4Fe–4S] iron–sulfur cluster.

Upon preliminary inspection of the sequences, XP 001467588.1 appeared to be a truncated form of CAC9521120.1 and was therefore excluded from subsequent analyses, leaving 11 full‐length flavoprotein sequences for further investigations. Additional structurally related flavoproteins identified during the subsequent structural modeling and docking analyses are reported in the following tables and figures associated with the comparative structural investigation.

### 3.2 Fold recognition tools suggest the presence of a natural grouping of flavoproteins in *L. infantum*

Fold‐recognition analyses performed with pGenThreader and I‐TASSER for each of the sampled *L. infantum* sequences yielded the results summarized in Table 2. These analyses revealed a clear structural grouping of the flavoproteins, separating them into trypanothione reductases (TRs), dihydrolipoamide dehydrogenases (DLDs), type II NADH dehydrogenases (NDH2s), small reductases (e.g., morphinone reductase and ene‐reductase), and mixed proteins such as dienoyl‐CoA reductase (deCoAR) and naphthoyl‐CoA reductase (nCoAR), which comprise both an NDH2‐like subunit and a small reductase subunit.

Structural superimposition using PyMOL confirmed a strong overlap between NDH2s and small reductases with the corresponding subunits of the mixed proteins 1ps9.pdb and 6qkr.pdb. In particular, crystallized small reductases (5n6g.pdb, 1gwj.pdb, and 6myw.pdb) superimposed closely with the reductase subunit of 1ps9.pdb (RMSD ≤1.1 Å), whereas representative DLD and NDH2 structures (1jeh, 5wed, 4g6h, 6awa, 2qae, 1fea, 5u8u, 1dxl, and 6uzi) aligned well with the NDH2‐like subunit (RMSD ≤4 Å). Conversely, structures 2rgh and 2rgo showed poor superposition with either subunit (RMSD ≥12 Å), suggesting that they represent structural outliers (data not shown).

To exclude the possibility that the crystallized small reductases represented truncated proteins, blastp searches were performed using the sequences of 5n6g, 1gwj, and 6myw against the nr database restricted to their respective taxa (*A. radiobacter*, *P. putida*, and *G. oxydans*), confirming that these proteins were crystallized in full length. Similarly, blastp analyses of the mixed proteins 6qkr.pdb and 1ps9.pdb demonstrated that both organisms encode comparable mixed enzymes in addition to the smaller reductases as identified above (respectively WP_174004742.1 and WP_177409672.1).

Finally, blastp searches against the *L. infantum* proteome confirmed the relationships suggested by fold recognition tools, with XP_003392329.1 emerging as the best match for 5n6g and 1gwj, XP_003392330.1 for 6myw, and XP_001468165.1 for both 1ps9 and 6qkr (Table 1).

Additional blastp analyses in *Homo sapiens* indicated that (1) mixed proteins and small reductases lack human counterparts, (2) *L. infantum* FAD/NAD(P)H‐dependent dehydrogenases show the highest level of similarity to human glutathione reductase, thioredoxin reductase, and DLD.

### 3.3 Sequence conservation of flavoproteins in *L. infantum* and other species

**FIGURE 2:**
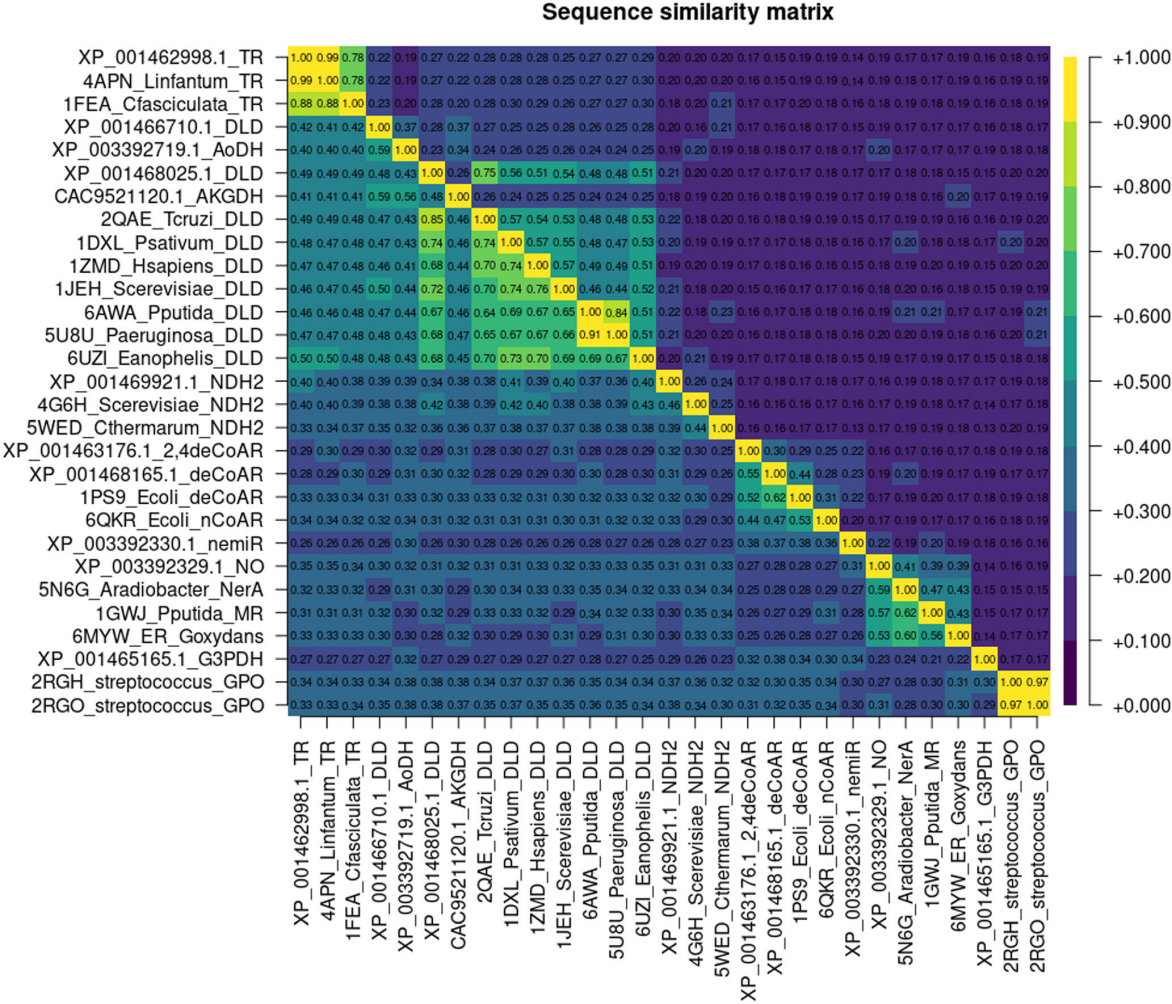
Sequence similarity and identity matrix of all *L. infantum* sequences discovered through sequence sampling and all protein crystals discovered through fold recognition sampling, including 4apn and 1zmd, found by keyword search on RCSB PDB. The upper triangular side of the matrix contains the sequence identity values, whereas the lower triangular side contains the sequence similarity values. AKGDH, α‐ketoglutarate dehydrogenase, AoDH, acetoin dehydrogenase; deCoAR, dienoylCoA reductase; DLD, dihydrolipoamide dehydrogenase; ER, ene‐reductase; G3PDH, glycerol‐3‐phosphate dehydrogenase; GPO, glycerol‐phosphate oxidase; MR, morphinone reductase; nCoAR, naphtoylCoA reductase; NDH2, type II NADH dehydrogenase; nemiR, n‐ethylmaleimide reductase; NO, NADH oxidase; TR, trypanothione reductase.

**FIGURE 3:**
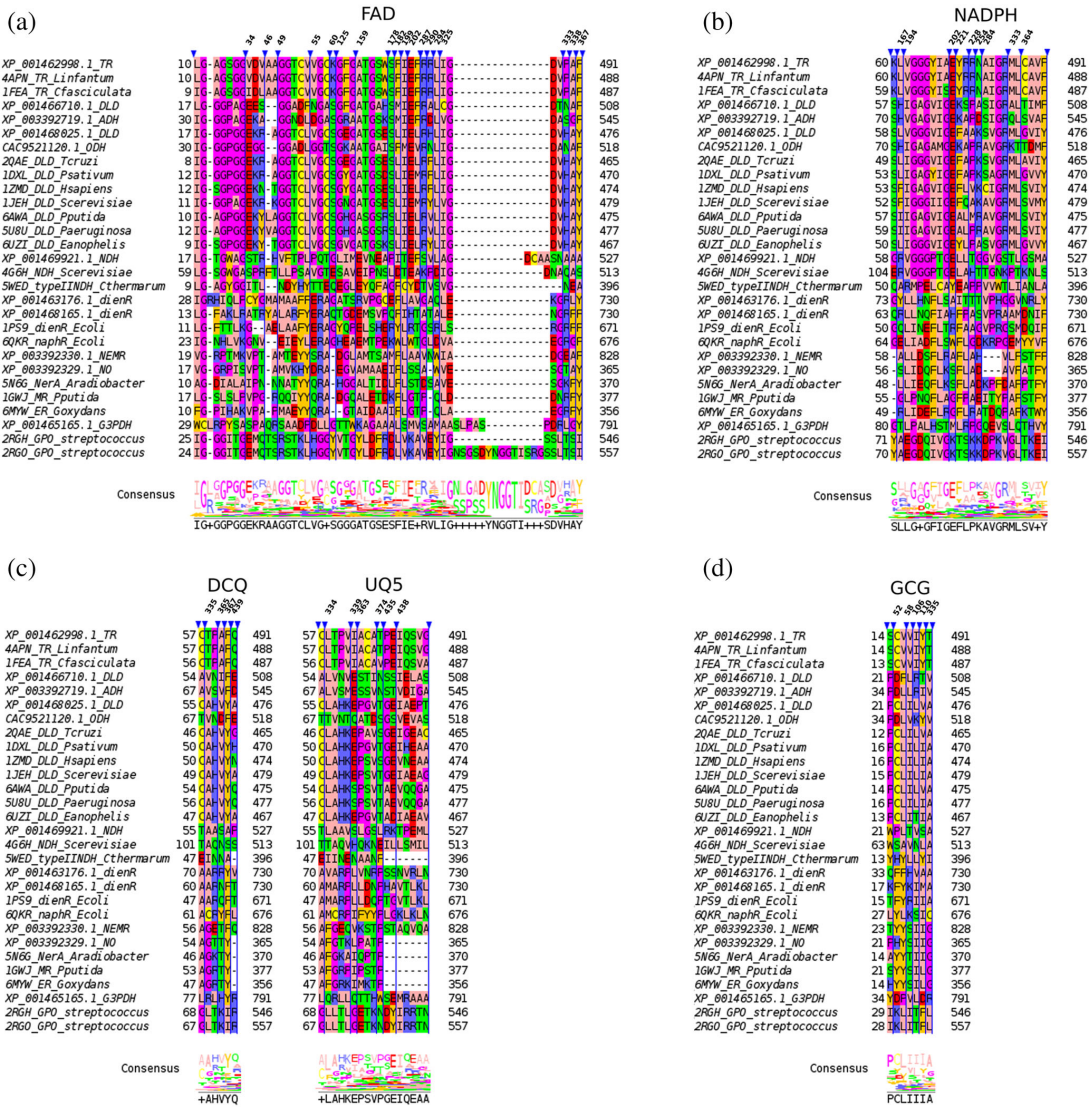
Conservation of cofactor binding sites. In panel (a), conservation of residues within 5 Å of FAD; in panel (b), conservation of residues within 5 Å of NADPH; in panel (c), conservation of residues within 5 Å of DCQ and UQ5; and in panel (d), conservation of residues within 5 Å of GCG. Numbering above the pictures is 4apn numbering. GCG corresponds to trypanothione, whereas UQ5 and DCQ are ubiquinone derivatives.

**FIGURE 4:**
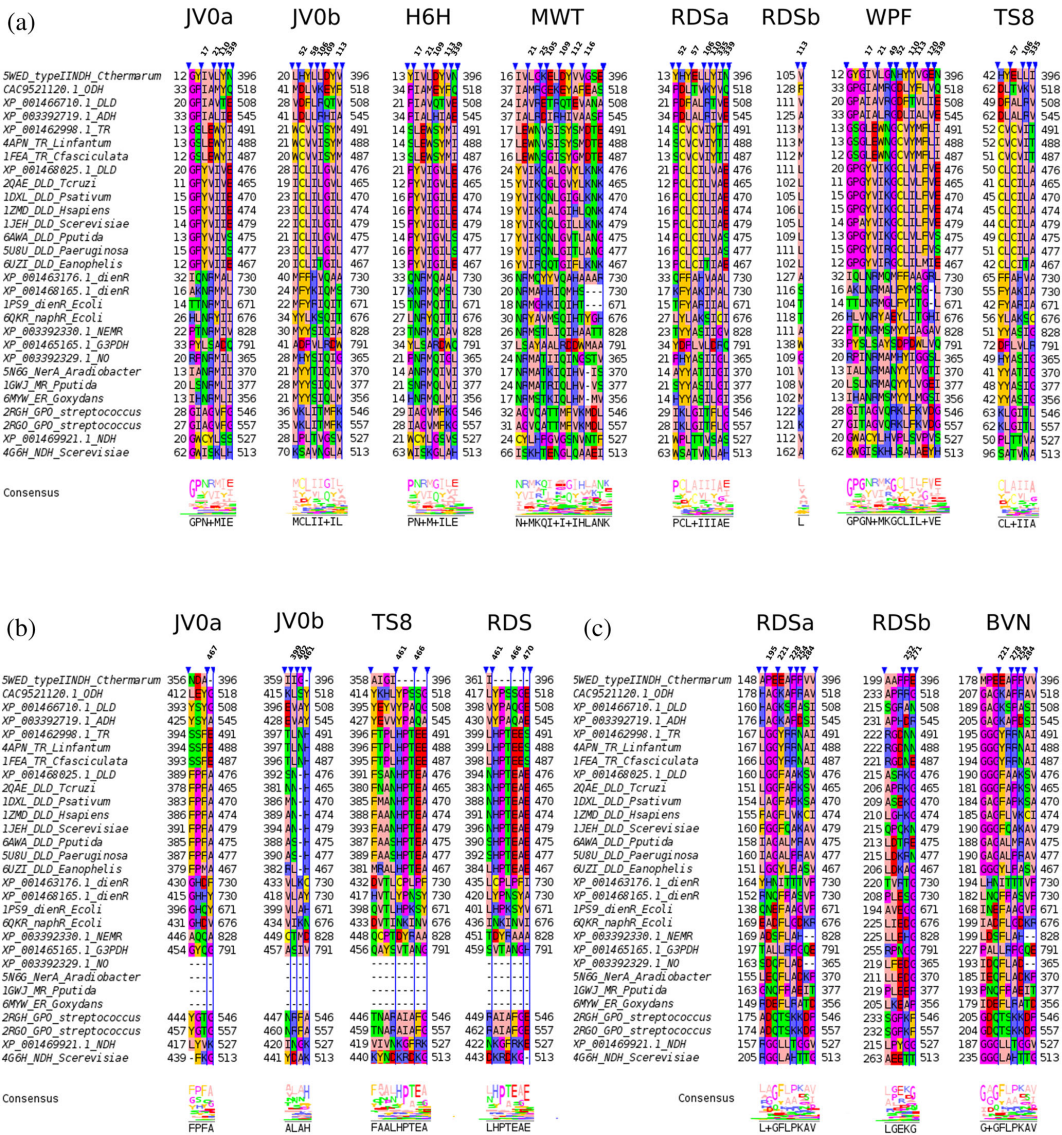
Conservation of inhibitor binding sites. In panel (a), conservation of residues within 5°A of ligands of site 1 (two JV0 poses, H6H, MWT, two RDS poses, WPF, TS8); in panel (b), conservation of residues within 5°A of ligands of site 2 (two JV0 poses, TS8, RDS); in panel (c), conservation of residues within 5°A of ligands of site 3 (two RDS poses, BVN). Numbering above the pictures is 4apn numbering.

Sequence similarity and identity derived from the multiple sequence alignment (MSA) are summarized in Figures 2, 3, 4. The clustering of TRs, DLDs, NDH2s, mixed proteins, small reductases, and glycerol‐phosphate‐interacting enzymes is readily apparent and closely mirrors the structural relationships observed upon superposition of the corresponding FAD and NAD(P)H cofactors.

**FIGURE 5:**
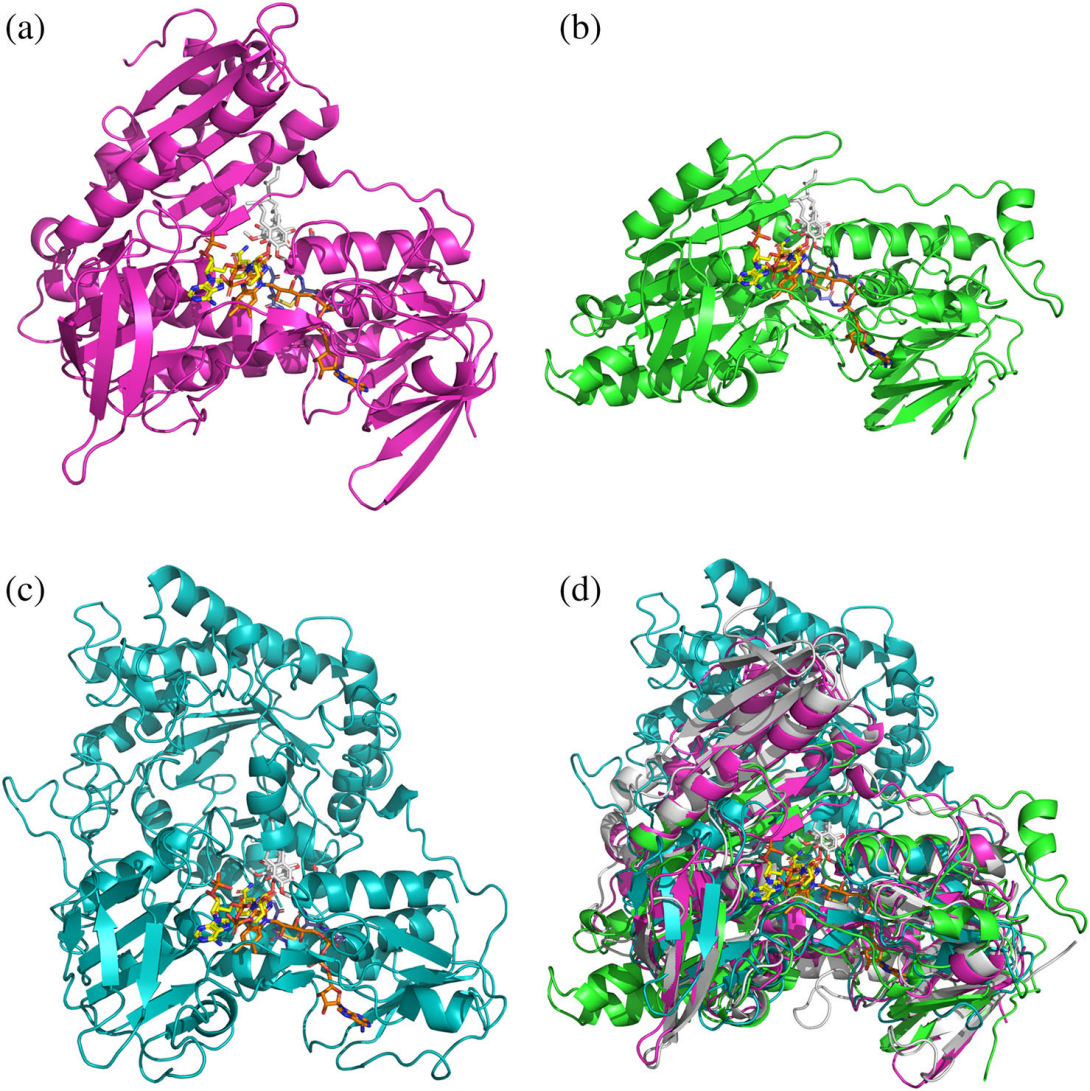
3D comparative models of *L. infantum* FAD/NAD(P)H‐dependent dehydrogenases. In panel (a), the 3D comparative model of XP 001468025.1 DLD in magenta, in panel (b) the XP 001469921.1 NDH2 model is depicted in light green, in panel (c) the XP 001468165.1 dienoylCoA reductase‐like protein (deCoAR) model depicted in teal, and in panel (d) a superimposition of all the models with LiTR (4apn.pdb), in white cartoon. The four panels also contain the cofactors tested in re‐docking analysis: FAD can be seen in orange, NAD(P)H in yellow, UQ5 and DCQ in white and trypanothione in pink (being the latter hidden behind the superimposed models of panel D).

**FIGURE 6:**
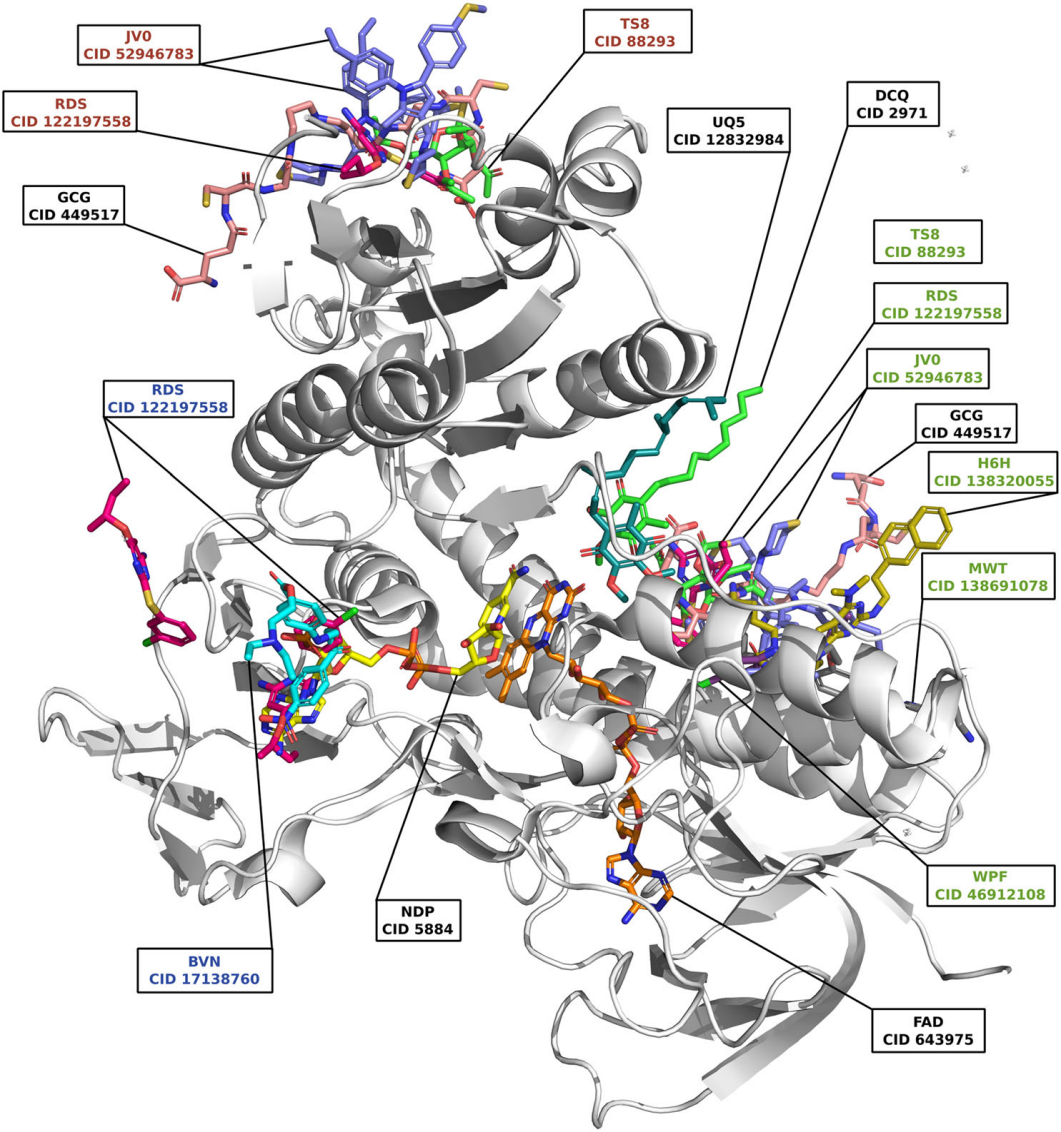
Structural representation of the inhibitors and cofactors analyzed in this study, illustrating their binding sites on *L. infantum* trypanothione reductase (PDB ID: 4apn). For each ligand, the PDB ID of the crystal structure from which the bound pose was derived and the corresponding PubChem CID are reported. For FAD and NADPH, the binding poses correspond to those available directly in the 4apn crystal structure. GCG denotes trypanothione, whereas UQ5 and DCQ represent ubiquinone derivatives.

The analysis of the residues involved in cofactor and inhibitor binding performed in Jalview revealed that amino acid conservation within cofactor‐binding regions is highest between TR and DLD, decreases in NDH2s, and is further reduced in deCoAR‐like proteins, although a residual degree of similarity is retained (Figures 5, 6).

Conservation patterns became more discriminative when inhibitor‐binding regions were analyzed (Figures 5, 6). In this case, a greater divergence was observed relative to the FAD/NAD(P)H‐dependent dehydrogenases when compared with α‐glycerophosphate oxidases and small reductases (Table 2). At the level of conserved motifs, the structurally related TRs and DLDs are still clearly distinguished (Figures 3, 4, 5, 6). NDH2s appeared as an intermediate group, bridging TR/DLD and deCoAR‐like proteins, whereas small reductases and α‐glycerophosphate oxidases displayed a more homogeneous conservation profile. In all cases, small reductases and glycerophosphate‐interacting enzymes remained clear outliers relative to the other sequences (Figure 2), justifying their exclusion from further analysis.

### 3.4 Analysis of TR structure, cofactors and inhibitor binding regions

A keyword‐based search of the RCSB Protein Data Bank resulted in the identification of several accessory cofactors previously reported to interact with structurally related FAD/NAD(P)H‐dependent dehydrogenases in four specific cofactor binding regions, as listed in Table 3 (Krämer et al., 2025; Oblong et al., 1994). Remarkably, UQ5 was crystallized only in complex with yeast NDH2 (PDB ID 4g73; Feng et al., 2012) and DCQ has exclusively been co‐crystallized with a sulfide:quinone oxydoreductase found in *A. aeolicus* (PDB ID 3hyw; Marcia et al., 2009). In addition, a small library of TR inhibitors was also collected, which appear to interact with three main binding pockets (sites 1–3), as summarized in Table 4, with their corresponding 2D chemical structures shown in Figure 1.

**TABLE 3:** In vitro estimated range of Km (reported in mM, where not differently indicated) values of known cofactors investigated in redocking and docking analyses on LiTR and other structurally related FAD/NAD(P)H‐dependent dehydrogenases.

Organism	Reference PDB_ID	FAD	NAD+/NADH	NADP+/NADPH	Ubiquinone/Decylubiquinone/Ubiquinol	Lipoamide	Dihydrolipoamide	Glutathione	Glutathione disulfide	Trypanothione (oxidized)	Thioredoxin	References	Brenda Database
*L. infantum* TR	4apn (4adw)			1.9 ± 0.7 μM						*13.5–30* μM		Angiulli et al. (2015), Hamilton et al. (2003), Ortalli et al. (2018), Turcano et al. (2018)	https://www.brenda‐enzymes.org/enzyme.php?ecno=1.8.1.12
S.cerevisiae NDH2	4g73		*0.0094–0.031*		Ubiquinone: 22.7 ± 1.0 μM							Yamashita et al. (2018)	https://www.brenda‐enzymes.org/enzyme.php?ecno=7.1.1.2
*C. thermarum NDH2*	4nwz (5wed)		29 ± μM									Blaza et al. (2017)	
*A. aeolicus* sulfide:quinone oxido reductase	3hyw				0.00216–0.005								https://www.brenda‐enzymes.org/enzyme.php?ecno=1.8.5.4
*dECoAR*				0.0025–0.215									https://www.brenda‐enzymes.org/enzyme.php?ecno=1.3.1.124
*H. sapiens* AIF	4bur	NA	0.007–0.27 /0.00005–1.1	552.9 ± 12	0.002–0.35	NA	NA	NA	NA		NA	Dipol et al. (2025)	
*H. sapiens* DLD	1zmd	NA	0.024–1.3/0.04–0.24	0.025–1.87	NA	0.05–5	0.027–43.6	NA	NA		NA	Dipol et al. (2025)	https://www.brenda‐enzymes.org/enzyme.php?ecno=1.8.1.4
*H. sapiens* GSR	1gra	0.00052–0.00078	0.017–1.12	0.008–0.22/0.0015–0.27	0.297/0.024–43	NA	NA	0.023–8.2	0.00171–2.31	0.5	NA	Dipol et al. (2025)	https://www.brenda‐enzymes.org/enzyme.php?ecno=1.8.1.7
*H. sapiens* TrxR1 (*P. falciparum*)	2j3n.pdb; (4j56.pdb)	NA	0.011–0.736	0.0004–4.5	NA	0.0019–5.59	NA	0.015	NA		0.00031–67.6	Dipol et al. (2025)	https://www.brenda‐enzymes.org/enzyme.php?ecno=1.8.1.9

*Note*: The Km values are reported in the forms indicated in the Brenda weblinks (under the Functional parameters/Km values menu), where other additional kinetic parameters can also be retrieved (https://www.brenda‐enzymes.org/). Representative PDB_IDs of the investigated protein are also reported.

Abbreviations: GCG corresponds to trypanothione, whereas UQ5 and DCQ are ubiquinone derivatives. For other PDB_IDs structurally related to those reported (Dipol et al., 2025; Trisolini et al., 2019),

**TABLE 4:** All identified inhibitors suitable for redocking in trypanothione reductase (TR) in *L. infantum* as obtained from RCSB PDB.

PDB ID	Annotation	Inhibitor	Site	𝐼𝐶_50_	References
4apn	*L. infantum* TR with inhibitor	JV0	1, 2	13.77 μM	Baiocco et al. (2013)
5ebk	*L. infantum* TR with inhibitor	RDS	1, 2, 3	29.43 ± 1.34 μM	Saccoliti et al. (2017)
6 t95	*L. infantum* TR with inhibitor	MWT	1	9.4 ± 1.6 μM	de Lucio et al. (2022)
6i7n	*L. infantum* TR with inhibitor	H6H	1	52.2 ± 1.8 μM	Revuelto et al. (2019)
2yau	*L. infantum* TR with inhibitor	TS8	1, 2	155 ± 35 𝑛𝑀	Ilari et al. (2012)
2wpf	*T. brucei* TR with inhibitor	WPF	1, 2	230 μM	Patterson et al. (2011)
6er5	*L. infantum* TR with inhibitor	BVN	3	7.5 ± 2.5 μM	Turcano et al. (2018)

Note: 𝐼𝐶_50_ values were obtained from the literature corresponding to each deposited crystal structure.

The sampled high affinity ligands bind to three main binding regions referred to as site 1, site 2, and site 3. Site 1 is in proximity to the FAD‐binding region, without directly overlapping the FAD‐binding pocket. Site 3 is structurally close to the NADPH binding region and partially overlaps with the NADPH adenine moiety binding pocket. In contrast, site 2 is located in a region more distant from the catalytic pocket (Figure 5). When multiple crystallographic poses of the same inhibitor were available, either within the same binding region or across distinct but spatially related sites, the binding free energy was calculated separately for each crystallized pose.

Based on the available crystallographic information, four cofactor‐binding regions were defined with reference to FAD, NADPH/NADH, trypanothione (GCG), glutathione (GSSG), and ubiquinone‐like molecules (Table 3). In addition, three inhibitor‐binding sites were delineated according to the spatial clustering of crystallized inhibitor poses (Table 4). This partitioning is illustrated in Figure 5.

### 3.5 Homology models of DLD, NDH2, and deCoAR

The *L. infantum* sequences selected for three‐dimensional modeling were XP_001468025.1, annotated as dihydrolipoamide dehydrogenase; XP_001469921.1, annotated as type II NADH dehydrogenase; and XP_001468165.1, annotated as dienoyl‐CoA reductase (Table 5). These annotations were consistent with the placement of each sequence within the clustering derived from reciprocal balstp and fold‐recognition analyses. Among the candidate templates identified in the previous steps, crystallized human proteins retrieved through keyword searches of the RCSB PDB were preferentially selected whenever available, to facilitate the evaluation of potential off‐target interactions or cross‐reactions of trypanothione reductase inhibitors with host human (or mammalian) enzymes. When no suitable human homolog was available, the best non‐human crystal structure was selected. For each *L. infantum* sequence, the final template was chosen by comparing the results obtained with pGenThreader and I‐TASSER.

**TABLE 5:** Structural comparison between the L. infantum FAD/NAD(P)H‐dependent dehydrogenase sequences and the most similar crystal structures identified through pGenThreader and I‐TASSER fold‐recognition analyses.

Sequence	PDB ID	pGenThreader annotation cofactor		RMSD (Å)	% identical residues	PDB ID	I‐TASSER annotation cofactor		RMSD (Å)	% identical residues
XP 001468025.1	5u8u	DLD	FAD	0.719	49.2	2qae	DLD	FAD	0.641	77.5
XP 001469921.1	5wed	NDH2	FAD	1.503	32.4	4g6h	NDH2	FAD	0.329	33.2
								NAI		
XP 001468165.1	6qkr	nCoAR	FAD	0.356	30.6	1 ps9	deCoAR	FAD	1.467	43.2
			FMN					FMN		

Note: RMSD values refer to the structural superimposition between the generated homology models and the corresponding crystal structures. The percentage of identical residues calculated by Jalview/ClustalW between the investigated sequences and the corresponding template is additionally reported. Annotations and cofactor information associated with the selected crystal structures were retrieved from the RCSB PDB database.

Abbreviations: deCoAR, dienoyl‐CoA reductase; DLD, dihydrolipoamide dehydrogenase; nCoAR, naphthoyl‐CoA reductase; NDH2, type II NADH dehydrogenase.

Accordingly, the crystallized human dihydrolipoamide dehydrogenase (1zmd.pdb) was selected as the primary template for modeling XP_001468025.1 based on the pGenThreader results (97% query coverage and 55% sequence identity). Although a closer homolog is represented by the *T. cruzi* DLD‐like protein (2qae.pdb), which exhibits higher sequence identity (95% coverage and 70% identity), the human enzyme was preferentially chosen to enable a direct and structurally consistent assessment of potential off‐target interactions of TR inhibitors with host flavoproteins. The use of a human template, therefore, reflects a deliberate choice, prioritizing biological relevance for selectivity analyses over maximal sequence identity, while maintaining sufficiently high coverage and identity to ensure reliable comparative modeling. For XP_001469921.1, the crystallized yeast NDH2 structure (4g6h.pdb) was selected based on the I‐TASSER results (81% coverage and 28% sequence identity; Table 1). Similarly, XP_001468165.1 was modeled using the *E. coli* dienoyl‐CoA reductase structure (1ps9.pdb), again identified by I‐TASSER as the best template (91% coverage and 44% identity; Table 1).

Following model construction, the quality of the generated 3D comparative models was assessed by superimposing each homology model onto its corresponding crystallized template using PyMOL. The resulting root‐mean‐square deviation (RMSD) values are reported in Table 5 Protein structural RMSD values were calculated after structural superimposition in PyMOL using the default atom‐matching procedures implemented in the align/super commands, based on the matched atoms included in the selected protein structures after iterative refinement. In all cases, RMSD values remained below 2 Å, indicating a satisfactory outcome of the comparative modeling procedure. The individual models, as well as their superposition with the *L. infantum* trypanothione reductase structure (4apn.pdb) and the relevant cofactors, are shown in Figure 5.

### 3.6 Redocking analysis on LiTR

To assess the reliability of the docking protocol and the suitability of the selected gridboxes, a systematic redocking analysis was performed on LiTR (PDB ID 4apn). High‐affinity ligands, cofactors, and inhibitors crystallized in complex with TR were re‐docked into their respective binding regions, which had been previously defined based on structural inspection of available crystal structures (Table 4). The list of the explored gridboxes with the corresponding gridbox parameters is reported in Table 6.

**TABLE 6:** Gridbox parameters of trypanothione reductase (PDB ID: 4apn) redocking.

Site	Ligand cofactor inhibitor		Center of gridbox	Grid points	4apn atom closer to the gridbox center
FAD	FAD		−55.309 34.454 −6.072	64 78 52	T51 OG1
NADPH	NADPH		−53.656 31.062 8.633	60 58 60	G197 CA
GCG/GSSG	GCGred		−39.278 21.984 −18.273	82 40 70	I106 CG1
	GCGox		−46.736 22.084 −12.987	50 40 42	Y110 OH
	GSSG		−46.547 22.798 −13.696	50 34 40	Y110 OH
UQ5/DCQ	UQ5		−48.942 16.609 −3.327	44 58 42	Q439 OE1
	DCQ		−46.309 16.593 −5.809	44 56 40	Q439 OE1
1		JV0	−45.691 23.829 −15.721	40 24 36	Y110 OH
		RDS	−46.945 22.645 −10.86	32 36 24	C52 SG
		MWT	−48.496 24.674 −23.885	58 36 56	M113 CB
		H6H	−49.563 18.211 −19.189	40 56 46	W21 NE1
		TS8	−44.938 22.91 −12.526	28 26 38	V58 CG2
		WPF	−51.163 24.099 −17.51	30 32 36	Y110 OH
2		JV0*	−29.671 3.338 17.082	44 44 36	E467 OE2
		JV0	−29.746 2.84 18.573	30 48 40	E467 OE2
		RDS	−28.697 3.533 11.026	32 42 28	H461 CE1
		TS8	−28.496 6.674 13.613	24 28 38	L399 CD1
3		RDS	−57.232 34.777 14.225	38 40 22	R222 NH2
		BVN*	−57.674 31.277 16.169	74 40 34	R222 NH2
		BVN	−58.155 32.121 16.149	40 42 40	R222 NH2

*Note*: GCG corresponds to trypanothione (GCGox, oxidized trypanothione, GCGred, reduced trypanothione), GSSG corresponds to oxidized glutathione, whereas UQ5 and DCQ are ubiquinone derivatives. As a further control, JV0* and BVN* show grid parameters employed for the docking performed on the dimeric protein.

Overall, re‐docking of FAD and NAD(P)H produced poses consistent with the experimentally observed conformations, supporting the robustness of the selected docking parameters. In contrast, reduced trypanothione could not be reliably re‐docked into the LiTR structure (4apn.pdb), nor into its crystal of origin (4adw.pdb), despite repeated attempts. This outcome is likely related to the high conformational flexibility of trypanothione (up to 27 for trypanothione [GCG] compared to the 13 rotatable bonds for FAD and NADPH), and the solvent‐exposed nature of its binding region, features that are known to limit the accuracy of docking‐based pose prediction for large cofactors, although the observed RMSD values fall within the range commonly reported for docking studies involving bulky cofactors (Vittorio et al., 2024).

Remarkably, to provide a further structural reference for trypanothione docking, we therefore considered the crystal structure of TR from *Trypanosoma cruzi* (1bzl.pdb; Bond et al., 1999) in complex with the oxidized trypanothione. This enzyme shares 68% of identical residues with *Li*TR (4apn.pdb) and displays an overall RMSD below 0.6 Å upon structural superposition with 4apn.pdb, supporting its suitability as a reference model for the oxidized trypanothione conformation used in our re‐docking analyses.

For those molecules that provided unsatisfactory redocking results (i.e., refRMS values greater than 2.5 Å as for RDS, BVN, and JV0); additional redocking attempts were carried out on the dimeric LiTR structure, if the tested ligand was known to bind at the monomer‐monomer interface, resulting in partial improvements in pose accuracy (Table 7). Remarkably, in all cases, reproduced binding poses are compatible with their crystallographic locations. Taken together, these results validate the docking setup for comparative analyses while highlighting the intrinsic limitations associated with large, flexible ligands and shallow binding pockets.

**TABLE 7:** Results of docking on 4apn.

PDB ID	Site	Ligand cofactor inhibitor		Binding energy (kcal/mol)	Predicted inhibition constant	RefRMS (Å)
4apn	FAD	FAD		−8.92	289.54 nM	2.36
	NADPH	NADPH		−9.35	140.53 nM	1.41
	UQ5/DCQ	UQ5		−6.42	19.61 μM	2.43
		DCQ		−4.87	267.29 μM	2.71
	GCG/GSSG	GCGox		−4.1	98.38 μM	2.57
		GCGred		1.28	NA	4.62
		GSSG		0.32	NA	2.67
	1		JV0	−5.63	74.38 μM	2.19
			RDS*	−7.29	4.57 μM	2.67
			RDS	−3.38	3.32 mM	3.41
			MWT	−6.47	19.45 μM	2.01
			H6H	−5.12	177.45 μM	2.48
			TS8	−0.82	251.59 μM	2.47
			WPF	−6.45	18.57 μM	2.05
	2		JV0*	−7.75	318.25 μM	2.14
			JV0	−1.72	55.2 mM	3.59
			RDS*	−6.83	9.84 μM	2.42
			RDS	−4.01	1.15 mM	3.72
			TS8	−2.24	22.98 μM	1.87
	3		RDS	−6.12	32.49 μM	1.55
			BVN*	−4.41	582.30 μM	2.75
			BVN	−4.67	380.06 μM	3.36

*Note*: The best binding energy and the related inhibition constants are reported together with the best refRMS obtained along the docking simulation. GCG corresponds to trypanothione (GCGox, oxidized trypanothione; GCGred, reduced trypanothione), GSSG corresponds to oxidized glutathione, whereas UQ5 and DCQ are ubiquinone derivatives. An asterisk (*) next to the ligand's name indicates that the ligand was docked on dimeric 4apn due to high refRMS (>3Å) when docked on the monomer alone. See also Table 6. Positive values in the “Binding energy” column or NA (not available) values in the “Inhibition constant” column indicate non‐binding due to high distance from protein residues or clashes with protein structures.

### 3.7 Preliminary selectivity analysis on human FAD/NAD(P)H‐dependent dehydrogenases

To explore potential off‐target interactions of TR ligands with host enzymes, a preliminary selectivity analysis was conducted by docking the same set of cofactors and inhibitors onto structurally related human FAD/NAD(P)H‐dependent dehydrogenases, including dihydrolipoamide dehydrogenase (DLD), glutathione reductase, thioredoxin reductase, and apoptosis‐inducing factor (AIF).

Cofactors such as FAD and NAD(P)H displayed comparable binding modes across all human enzymes analyzed, reflecting the conserved architecture of their cofactor‐binding sites. In contrast, inhibitor docking revealed marked variability. Several compounds showed predicted affinities similar to or even higher than those observed for TR, particularly in the case of human DLD. Notably, RDS, JV0, and WPF consistently produced stable docking solutions in both LiTR and DLD, suggesting a potential for cross‐reactivity with host enzymes (Tables 7, 8). For other ligands, predicted affinities were reduced, or binding poses were less stable in human proteins, indicating a degree of selectivity toward the parasite enzyme. Although docking‐derived binding parameters cannot be directly equated with experimentally measured values, relative comparisons of estimated inhibition constants (K_i_) can provide useful insights into ligand selectivity (Cafferati Beltrame et al., 2024; Cafferati Beltrame et al., 2025; Goodsell et al., 1996; Todisco et al., 2016) (Tables 7, 8).

**TABLE 8:** Results of docking on 1zmd chain C and 4bur chain A.

PDBID	Site	Cofactor	Inhibitor	Binding energy (kcal/mol)	Inhibition constant	RefRMS (Å)
1zmd	FAD	FAD		−4.68	368.41 μM	2.32
	NADH	NADH		−7.47	3.35 μM	1.81
	UQ5/DCQ	UQ5		−4.74	337.99 μM	2.73
		DCQ		−4.12	955.94 μM	2.25
	GCG/GSSG	GCGred		1.43	NA	3.62
		GCGox		−9.17	188.80 nM	2.65
		GSSG		0.36	NA	2.66
	1		JV0	−3.83	1.56 mM	2.65
			RDS	−3.82	1.59 mM	2.48
			MWT	−2.92	7.27 mM	4.82
			H6H	−2.68	10.8 mM	3.35
			TS8	−1.84	44.47 mM	2.52
			WPF	−4.88	264.47 μM	4.57
	2		JV0	−5.01	212.64 μM	3.07
			RDS	−5.40	110.76 μM	3.30
			TS8	−3.53	2.6 mM	1.91
	3		RDS	−6.20	28.76 μM	2.41
			BVN	−3.02	6.14 mM	3.49
4bur	FAD	FAD		−12.63	553.23 pM	2.15
	NADH	NADH		−10.58	17.57 nM	1.76
	UQ5/DCQ	DCQ		−4.62	409.88 μM	2.68
		UQ5		−4.52	488.13 μM	2.56
	GCG/GSSG	GCGred		3.47	NA	4.52
		GCGox		−9.10	214.49 nM	3.54
		GSSG		−2.02	33.01 mM	3.51
	1		JV0	−4.03	1.12 mM	3.06
			RDS	−3.63	2.18 mM	4.58
			MWT	−6.21	28.12 μM	3.92
			H6H	−2.59	12.71 mM	3.47
			WPF	48.12		3.16
			TS8	−2.1	28.97 mM	2.59
	2		JV0	−0.29	610.58 mM	4.51
			RDS	0.48		4.70
			TS8	2.32		2.69
	3		BVN	−2.85	8.13 mM	3.90
			RDS	−4.4	595.87 μM	3.67

*Note:* While best binding energy and best inhibition constants are obtained from the same pose, the best refRMS may be retrieved from poses other than the first. GCG corresponds to trypanothione (GCGox, oxidized trypanothione, GCGred, reduced trypanothione), whereas UQ5 and DCQ are ubiquinone derivatives. The commercial name of TS8 is auranofin. High refRMS values (>2.65Å), positive values in the “Binding energy” column, or NA (not available) values in the “Inhibition constant” column indicate non‐binding due to high distance from protein residues or clashes with protein structures.

Predicted binding affinities varied considerably depending on the investigated ligand, binding region, and enzyme model. While several compounds, including TS8, BVN, MWT, RDS, H6H, and WPF, produced docking poses associated with estimated inhibition constants ranging from the micromolar to the millimolar scale across LiTR and related human flavoproteins, these computational predictions were not always fully consistent with the currently available biochemical data (Tables 3, 4, 5, 6, 7, 8, 9, 10). Nevertheless, comparative docking analyses remained informative for identifying relative trends in ligand selectivity and potential cross‐reactivity among structurally related FAD/NAD(P)H‐dependent dehydrogenases (Tables 3, 4, 5, 6, 7, 8, 9, 10). Docking analyses on DLD showed a trend similar to the one observed for LiTR (except for H6H ligand, which shows a higher predicted affinity for LiTR compared to DLD). Conversely, docking runs performed on AIF revealed a marked decrease in binding affinity for WPF at site 1, while the remaining compounds exhibited only minor variations (Table 8). A trend with a few variations was also observed for GSR, most notably for MWT at site 1 (Table 9). In the case of TrxR1, JV0 showed an increased predicted affinity at site 2, whereas H6H displayed a reduced binding capability at site 1 (Table 9).

**TABLE 9:** Results of docking on 1grb chain A and 2j3n chain D.

PDBID	Site	Cofactor	Inhibitor	Binding energy (kcal/mol)	Inhibition constant	RefRMS(Å)
1grb	FAD	FAD		−9.51	106.22 nM	2.68
	NADPH	NADPH		−8.39	708.06 nM	2.69
	UQ5/DCQ	DCQ		−5.32	126.88 μM	4.22
		UQ5		−7.01	7.29 μM	3.23
	GCG/GSSG	GCGred		4.98	NA	3.41
		GCGox		−8.90	300.48 nM	2.02
		GSSG		−3.01	6.22 mM	2.29
	1		JV0	−3.68	2.02 mM	2.58
			RDS	−4.02	1.12 mM	4.41
			MWT	−2.27	21.52 mM	4.51
			H6H	−2.74	9.79 mM	4.22
			WPF	−4.56	456.06 μM	4.11
			TS8	−1.49	81.28 mM	2.35
	2		JV0	−2.16	26.25 mM	3.91
			RDS	−3.71	1.92 mM	4.46
			TS8	−2.53	13.91 mM	2.57
	3		BVN	−4.24	780.82 μM	3.32
			RDS	−6.1	33.57 μM	5.26
2j3n	FAD	FAD		−11.48	3.84 nM	2.39
	NADPH	NADPH		−9.88	56.99 nM	2.26
	UQ5/DCQ	DCQ		−4.55	464.49 μM	3.69
		UQ5		−6.64	13.55 μM	3.96
	GCG/GSSG	GCGred		4.71	NA	3.52
		GCGox		−8.64	463.57 nM	2.76
		GSSG		−1.49	80.46 mM	2.60
	1		JV0	−4.43	566.32 μM	2.79
			RDS	−4.29	720.50 μM	4.86
			MWT	−4.7	361.44 μM	3.51
			H6H	−3.99	1.19 mM	4.12
			WPF	−4.23	789.19 μM	3.63
			TS8	−0.97	193.36 mM	2.54
	2		JV0	−6.31	23.60 μM	3.27
			RDS	−5.4	109.79 μM	4.58
			TS8	−3.35	3.49 mM	2.45
	3		BVN	−4.25	764.85 μM	3.84
			RDS	−7.83	1.84 μM	2.04

*Note*: While best binding energy and best inhibition constants are obtained from the same pose, the best refRMS may be retrieved from other poses than the first. GCG corresponds to trypanothione (GCGox, oxidized trypanothione, GCGred, reduced trypanothione), whereas UQ5 and DCQ are ubiquinone derivatives. The commercial name of TS8 is auranofin. High refRMS values (>2.65 Å), or positive values in the “Binding energy” column, or NA (not available) values in the “Inhibition constant” column indicate non‐binding due to high distance from protein residues or clashes with protein structures.

Although predicted binding affinities should be interpreted exclusively in a relative manner—that is, to compare binding trends rather than to provide absolute quantitative estimates—the present analysis establishes a robust and transferable computational framework for future docking studies aimed at identifying novel ligands targeting trypanothione reductase. This approach is applicable both to the development of antileishmanial agents and to the investigation of other pathological conditions involving FAD/NAD(P)H‐dependent dehydrogenases. Importantly, the results also emphasize the need to explicitly account for host flavoproteins when assessing the selectivity and potential off‐target liabilities of TR inhibitors. To facilitate comparative interpretation of potential selectivity and cross‐reactivity trends across parasite and human flavoproteins, the relative differences in best predicted binding energies between LiTR and the investigated human homologs are summarized in Table 10. Comparative inspection of the ΔBE values summarized in Table 10 highlighted that compounds such as JV0, H6H, MWT, and WPF generally displayed more favorable predicted binding energies toward LiTR than toward the investigated human flavoproteins. In contrast, TS8 and BVN exhibited more comparable docking trends across parasite and host enzymes, suggesting possible interaction with human flavoproteins.

**TABLE 10:** Comparative docking trends of investigated inhibitors across LiTR and human FAD/NAD(P)H‐dependent dehydrogenases.

Ligand	Best BE on LiTR (4apn)	Best BE on DLD (1zmd)	ΔBE DLD–LiTR	Best BE on AIF (4bur)	ΔBE AIF–LiTR	Best BE on GSR (1grb)	ΔBE GSR–LiTR	Best BE on TrxR1 (2j3n)	ΔBE TrxR1–LiTR	Predicted comparative trend
JV0	−7.75	−5.01	+2.74	−4.03	+3.72	−3.68	+4.07	−6.31	+1.44	LiTR‐favored
RDS	−7.29	−6.20	+1.09	−4.40	+2.89	−6.10	+1.19	−7.83	−0.54	Broad flavoprotein binder
MWT	−6.47	−2.92	+3.55	−6.21	+0.26	−2.27	+4.20	−4.70	+1.77	Moderately LiTR‐favored
H6H	−5.12	−2.68	+2.44	−2.59	+2.53	−2.74	+2.38	−3.99	+1.13	LiTR‐favored
TS8 (auranofin)	−2.24	−3.53	−1.29	−2.10	+0.14	−2.53	−0.29	−3.35	−1.11	Potential cross‐reactivity
WPF	−6.45	−4.88	+1.57	NA	NA	−4.56	+1.89	−4.23	+2.22	LiTR‐favored
BVN	−4.67	−3.02	+1.65	−2.85	+1.82	−4.24	+0.43	−4.25	+0.42	Moderate cross‐reactivity

*Note*: For each ligand, the best binding energy (BE, kcal/mol) obtained on LiTR (4apn) and on the investigated human homologs is reported. ΔBE values were calculated as follows: ΔBE = BE(human homolog) − BE(TR). Positive ΔBE values indicate weaker predicted binding toward the human homolog compared with TR, whereas negative ΔBE values indicate more favorable predicted binding toward the human homolog. Because docking‐derived energies were interpreted comparatively rather than as absolute predictors of affinity, the reported values should be considered qualitative indicators of potential selectivity or cross‐reactivity trends.

### 3.8 Selectivity and docking analysis on homology models of *L. infantum* FAD/NAD(P)H‐dependent dehydrogenases structurally related to LiTR

To further evaluate intra‐parasite selectivity, docking analyses within the four cofactor binding regions (Table 3) and the seven inhibitor binding regions highlighted in *Li*TR (sites 1–3, Table 4), were extended to the corresponding binding regions in the homology models of *L. infantum* flavoproteins structurally related to TR, namely type II NADH dehydrogenase (NDH2), dihydrolipoamide dehydrogenase (DLD), and dienoyl‐CoA reductase (deCoAR) (Table 5). These models were generated as described in the Methods section and structurally aligned to TR to allow consistent gridbox transfer after their superimposition on LiTR. Docking results revealed that TR inhibitors exhibited variability in the affinity toward NDH2 and deCoAR compared to LiTR. In particular, XP_001469921.1 NDH2 generally displayed intermediate behavior, with docking results suggesting partial compatibility with some TR ligands but weaker binding overall. On the XP 001469921.1 NDH2 model, trypanothione bonded very weakly. The ubiquinone derivatives UQ5 and DCQ bound solidly to their site, but many inhibitors completely lost their ability to bind the protein: among these, only MWT (site 1) retained a good estimated inhibition constant (Table 10). These results suggest different degrees of enzyme selectivity among the investigated inhibitors, within the parasite flavoprotein repertoire. This variability in binding affinity should be explored to develop new higher affinity selective ligands. Binding site 2 on NDH2 and binding sites 1 and 2 on deCoAR (in combination with GCG, UQ5, and DCQ) could not be explored in the docking analyses due to enzyme‐specific structural constraints. In particular, bulky or sterically hindered regions prevented reliable docking simulations for these specific ligand–enzyme combinations.

Conversely, ligands that showed strong predicted binding to LiTR and human DLD often also retained appreciable affinity for the *L. infantum* DLD model, reinforcing the notion that selectivity between parasite TR and parasite DLD may be limited for certain chemical scaffolds.

For this model, only WPF (site 1), JV0 (site 2) and RDS (site 2) retained a notable affinity, with an estimated *Ki* lower than 500 μM. Lastly, in the XP_001468165.1 deCoAR model, binding of cofactors was satisfactory, and the estimated constant of inhibition of RDS (site 3) remained remarkable (Table 11). Overall, the re‐docking experiment suggests the existence of off‐target interactions of the investigated TR inhibitors with other flavoproteins present in *L. infantum*. Conversely, the highlighted differences among parasite flavoproteins should be explored to make ligand recognition more selective.

**TABLE 11:** Results of docking on the XP 001468025.1 model, built using 1zmd (DLD) as a template, results of docking on the XP 001469921.1 model, built using 4g6h (NDH2) as a template, and results of docking on the XP 001468165.1 model, built using 1ps9 (deCoAR) as a template.

Modeled sequence	Site	Cofactor	Inhibitor	Binding energy (kcal/mol)	Inhibition constant	RefRMS (Å)
XP 001468025.1 (DLD‐like)	FAD	FAD		−7.52	3.05 μM	1.16
	NADH	NADH		−7.77	2.01 μM	3.44
	UQ5/DCQ	UQ5		−4.55	463.67 μM	2.03
		DCQ		−3.98	1.21 mM	2.61
	GCG/GSSG	GCGred		1.22	NA	3.45
		GCGox		−8.76	420.01 nM	3.01
		GSSG		−3.01	6.22 mM	3.29
	1		JV0	−3.12	5.16 mM	2.70
			RDS	−4.11	965.49 μM	3.94
			MWT	−2.4	17.42 mM	5.49
			H6H	−2.21	23.94 mM	4.15
			TS8	−1.01	182.61 mM	2.45
			WPF	−3.64	2.16 μM	4.21
	2		JV0	−5.08	190.02 μM	3.11
			RDS	−4.74	334.98 μM	3.07
			TS8	−2.73	9.96 mM	1.98
	3		RDS	−4.74	949.47 μM	3.07
			BVN	−4.39	603.29 μM	3.17
XP 001469921.1 (NDH2‐like)	FAD	FAD		−9.03	151.39 nM	2.5
	NADH	NADH		−7.83	1.82 μM	2.47
	GCG/GSSG	GCGred		−2.27	21.58 mM	12.97
		*GCGox		−8.93	386.65 nM	8.82
		#GGCox		78.03	NA	3.18
		GSSG		NA	NA	NA
	UQ5/DCQ	UQ5		−7.09	6.36 μM	2.03
		DCQ		−4.93	241.57 μM	3.49
	1		JV0	170.59	NA	2.83
			RDS	−1.73	53.67 mM	3.68
			MWT	−4.74	334.92 μM	3.95
			H6H	−3.06	5.71 mM	3.66
			TS8	567.97	NA	2.1
			WPF	20.17	NA	3.76
	3		RDS	−3.39	3.3 mM	2.13
			BVN	−1.76	51.25 mM	3.55
XP 001468165.1 (deCoAR‐like)	FAD	FAD		−10.48	20.76 nM	2.53
	NADPH	NADPH		−9.58	95.53 nM	2.45
	3		BVN	−4.34	655.8 μM	3.00
			RDS	−4.75	329.02 μM	2.18

*Note*: GCG corresponds to trypanothione, GSSG corresponds to oxidized glutathione, whereas UQ5 and DCQ are ubiquinone derivatives. High refRMS values (>2.65 Å), or positive values in the “Binding energy” column, or NA (not available) values in the “Inhibition constant” column indicate non‐binding due to high distance from protein residues or clashes with protein structures. In this case, the grid box used for docking GCGox (see Table 6) yielded only clustered poses with positive binding energies. To assess whether this outcome was influenced by the grid box definition, GCGox was re‐docked using the grid box previously defined for GCGred (Table 6), obtaining the binding results reported here below.

### 3.9 Docking of known FAD/NAD(P)H‐dependent dehydrogenase inhibitors on TR

**FIGURE 7:**
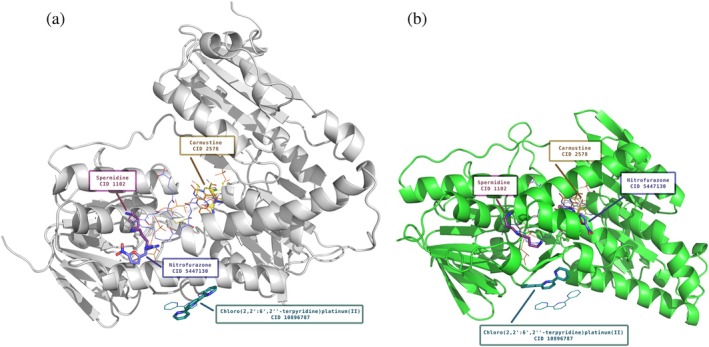
Structural representation of selected inhibitors and cofactors mapped onto *L. infantum* trypanothione reductase (LiTR, PDB ID: 4apn; panel a) and *C. thermarum* type II NADH dehydrogenase (CtNDH2, PDB ID: 5wed; panel b). For each ligand, the PDB ID of the crystal structure of origin and the corresponding PubChem CID are indicated where applicable. FAD and NAD(P)H coordinates were taken directly from their respective crystal structures. Spermidine (violet sticks) and nitrofurazone (blue sticks) localize within the trypanothione (GCG; light violet lines) binding region. Carmustine (light orange sticks) is positioned in correspondence with the NAD(P)H binding region (yellow lines). Chloro(2,2′:6′,2″‐terpyridine)platinum is shown either in its crystallized conformation (green lines; PDB ID: 1typ) or in the docked pose (green sticks), depending on the structure analyzed.

To further probe the breadth of LiTR ligand recognition, a set of known inhibitors of FAD/NAD(P)H‐dependent dehydrogenases other than TR, including chloro‐terpyridine platinum (structurally related to TPT, from 2zz.pdb, https://go.drugbank.com/drugs/DB01912; Dipol et al., 2025; Lo et al., 2009; Pierri & Pierri, 2026), carmustine (https://go.drugbank.com/drugs/DB00262; Arscott et al., 1997; Dipol et al., 2025; Pierri & Pierri, 2026), nitrofurazone (https://go.drugbank.com/drugs/DB00336; Dipol et al., 2025; Exertier et al., 2024; Pierri & Pierri, 2026), already crystallized in complex with other proteins, such as azoreductases (3r6w.pdb; Ryan et al., 2011) and nitroreductases (1yki.pdb; Race et al., 2005), and spermidine (SPD, from *Crithidia fasciculata* TR 1typ.pdb, https://go.drugbank.com/drugs/DB03566; Bailey et al., 1993), was docked onto LiTR and CtNDH2 (Figure 7). The selected compounds target diverse enzymes and binding sites, including allosteric regions and cofactor‐binding pockets.

Docking simulations were performed using binding‐site‐specific gridboxes derived from crystallographic data, except in cases where the binding site had not been previously characterized. Under these conditions, two out of the four compounds exhibited weak binding to LiTR, consistent with their reported specificity for other enzyme families. Notably, some ligands produced docking poses compatible with LiTR binding regions, suggesting possible cross‐reactivity or previously unrecognized interaction modes (Table 12). PX‐12 (https://go.drugbank.com/drugs/DB05448; Dipol et al., 2025; Ramanathan et al., 2007; Pierri & Pierri, 2026, yet to be crystallized) was excluded from this analysis due to the absence of a proposed binding site, which precluded meaningful docking simulations. Overall, this exploratory analysis complements the redocking and selectivity studies by illustrating the extent to which LiTR can accommodate structurally diverse ligands within its binding landscape.

**TABLE 12:** Results of docking chloro‐terpyridine platinum, carmustine, nitrofurazone, spermidine and their respective reference compounds on LiTR (PDB ID 4apn) and CtNDH2 (PDB ID 5wed).

Crystal	Site	Compound	Binding energy (kcal/mol)	Inhibition constant	RefRMS (A)
4apn	TPT	Chloro(2,2′:6′,2″‐terpyridine)platinum	−1.25	5.13 μM	NA
		TPT	−1.31	10.33 μM	2.48
	NADPH	Carmustine	−5.14	169.96 μM	NA
		NADPH	−9.35	140.53 nM	1.41
	GCG	Nitrofurazone_mon_	−3.44	3.00 mM	NA
		Nitrofurazone_dim_	−7.12	6.01 μM	NA
		GCG_ox_	−4.1	98.38 μM	2.57
		GCG_red_	1.28	NA	4.62
	SPD	SPD	−2.46	215.83 μM	2.14
5wed	TPT	Chloro(2,2′:6′,2″‐terpyridine)platinum	−5.03	206.80 μM	NA
		TPT	−5.10	183.73 μM	4.55
	NADPH	Carmustine	−4.84	282.25 μM	NA
		NADPH	−6.31	23.83 μM	4.28
	GCG	Nitrofurazone_mon_	−4.68	373.33 μM	NA
		^#^GCG_ox_	3.12 × 10^3^	NA	2.18
		*GCG_ox_	−9.88	57.15 nM	7.99
		GCG_red_	−0.89	223.34 mM	5.13
	SPD	SPD	−2.48	15.19 mM	3.05

*Note*: Chloro(2,2′:6′,2″‐terpyridine)platinum, structurally related to TPT crystallized in complex with thioredoxin reductase (PDB ID 2zzb). For nitrofurazone, proposed to bind at the GCG binding regiuons, the values of both reduced (GCGred) and oxidized trypanothione (GCGox) docking scores (binding energies) were compared. For each site, the test compound is reported first (e.g., carmustine binding energy in the NADPH binding region is compared to NADPH binding energ, etc.). SPD corresponds to the Chemical ID for spermidine in the protein data bank (PDB ID 1typ), it was just docked in 4apn.pdb or 5wed.pdb after re‐docking with 1typ.pdb. High refRMS values (>2.65 Å), or positive values in the “Binding energy” column, or NA (not available) values in the “Inhibition constant” column indicate non‐binding due to high distance from protein residues or clashes with protein structures. #In this case, the grid box used for docking GCGox (see Table 6) yielded only clustered poses with positive binding energies. To assess whether this outcome was influenced by the grid box definition, GCGox was re‐docked using the grid box previously defined for GCGred (Table 6), obtaining the binding results reported here below.

### 3.10 Expression and purification of LiTR and CtNDH2

**FIGURE 8:**
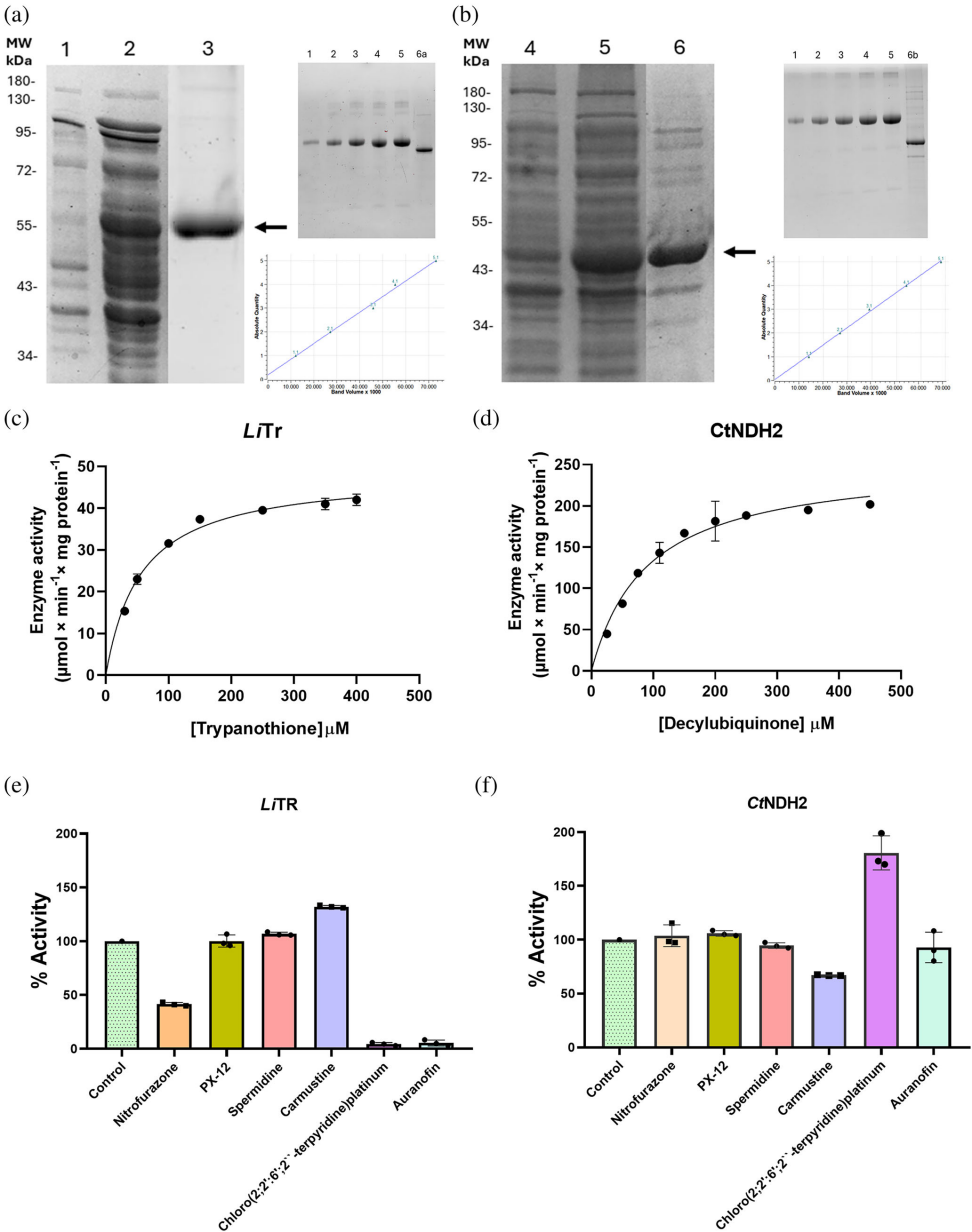
Expression, purification, kinetic characterization, and activity profiling of recombinant *L. infantum* trypanothione reductase (LiTR) and *C. thermarum* type II NADH dehydrogenase (CtNDH2). (a, b) SDS–PAGE analysis of recombinant protein expression and purification, visualized by Coomassie Brilliant Blue staining. (a) Lanes: supernatant of *E. coli* cell lysates expressing LiTR before (1) and after IPTG induction (2); purified LiTR (3). (b) Lanes: supernatant of *E. coli* cell lysates expressing CtNDH2 before (4) and after IPTG induction (5); purified CtNDH2 (6). Black arrows indicate the recombinant proteins. SDS–PAGE‐based quantification of purified proteins is also reported in panels (a) and (b). Lanes 1–5 contain increasing amounts of bovine serum albumin (BSA; 1–5 μg) used to generate the calibration curve. Lane 6a contains purified LiTR (1 μL), and lane 6b contains purified CtNDH2 (5 μL). Standard curves obtained by densitometric analysis using Image Lab software, demonstrating a linear relationship between band intensity and protein amount (R^2^ values indicated) are also reported. (c, d) Michaelis–Menten kinetic analysis of LiTR with trypanothione (c) and CtNDH2 with decylubiquinone (d), determined by measuring initial reaction rates at increasing substrate concentrations. (e, f) Effect of selected compounds on enzymatic activity. Relative activity of LiTR (e) and CtNDH2 (f) measured in the presence of test compounds at 100 μM, except for nitrofurazone, which was tested on CtNDH2 at 50 μM. The first bar in each plot represents the negative control (vehicle only), set to 100% activity. Auranofin was included as a positive control. Data represent the mean of two independent experiments; each performed in triplicate.

Recombinant *Li*TR and CtNDH2 were heterologously expressed in *E. coli* BL21(DE3) and C41(DE3), respectively, following the procedures described in the Materials and Methods section. As shown in Figure 8, both enzymes were efficiently recovered from the soluble fraction of the bacterial lysates and purified to near homogeneity.

Quantitative analysis by SDS–PAGE densitometry indicated final protein concentrations of 3.06 μg μL^−1^ (5.9 mg L^−1^ of culture) for *Li*TR and 0.35 μg μL^−1^ (1.6 mg L^−1^ of culture) for CtNDH2, which were subsequently used for enzymatic characterization and inhibition assays.

### 3.11 Kinetic characterization of recombinant LiTR and CtNDH2

The catalytic properties of the purified recombinant enzymes were assessed by measuring initial reaction rates over increasing substrate concentrations, ranging from 30 to 150 μM for trypanothione (LiTR) and from 75 to 250 μM for decylubiquinone (CtNDH2). In both cases, the enzymes followed Michaelis–Menten kinetics (Figure 8), allowing determination of the apparent Michaelis–Menten constants (Km) and maximum velocities (Vmax).

LiTR displayed an apparent Km for trypanothione of 48.55 μM and a Vmax of 56.05 μmol min^−1^ mg^−1^. The experimentally determined Km value for trypanothione was moderately higher than some values previously reported for LiTR in the literature (Table 3), possibly reflecting differences in recombinant enzyme preparation, substrate handling, or assay conditions among independent studies. For CtNDH2, the Km for decylubiquinone was 90.65 μM, with a Vmax of 254.45 μmol min^−1^ mg^−1^. These parameters indicate that both recombinant enzymes are catalytically active and display substrate affinities and turnover rates consistent with previously reported values for related systems under comparable assay conditions.

### 3.12 Modulation of enzymatic activity by selected compounds

To evaluate the effects of selected compounds on enzyme activity, LiTR and CtNDH2 were assayed in the presence of nitrofurazone, PX‐12, spermidine, and carmustine. Reactions were carried out at a fixed substrate concentration of 50 μM, chosen as a compromise between the Km values reported in the literature for LiTR (Hamilton et al., 2003; Ortalli et al., 2018; Turcano et al., 2018) and CtNDH2 (Heikal et al., 2014) and those determined experimentally in this study (Figure 8). Chloro(2,2′:6′,2″‐terpyridine)platinum and auranofin, two well‐established LiTR inhibitors, were included as positive controls and tested on both enzymatic systems. Prior to initiation of the reaction, enzymes were pre‐incubated with each test compound, dissolved in DMSO, ultrapure water, or ethanol as appropriate, according to methods described in Section 2.11. Reactions were initiated by the addition of saturating concentrations of NADPH (100 μM) for LiTR or NADH (200 μM) for CtNDH2. Enzymatic activities are reported in Figure 8 as relative activity (%) compared to the negative control, which was set to 100%.

In *Li*TR assays, nitrofurazone (100 μM) caused a marked inhibition, reducing enzyme activity to 41.52%. In contrast, PX‐12 and spermidine (100 μM) had no significant effect, yielding relative activities of 101.37% and 107.24%, respectively. Notably, carmustine (100 μM) acted as a positive modulator, increasing *Li*TR activity to 132.38%. As expected, the positive controls chloro(2,2′:6′,2″‐terpyridine)platinum and auranofin strongly inhibited *Li*TR, with residual activities of 4.44% and 5.76%, respectively.

When tested against CtNDH2, nitrofurazone (50 μM) and PX‐12 (100 μM) produced negligible effects, with relative activities of 106.43% and 103.97%, respectively. Similarly, spermidine and auranofin (100 μM) had only minor effects, maintaining enzyme activity at 94.99% and 94.32%. In contrast, carmustine (100 μM) significantly inhibited CtNDH2, reducing activity to 66.72%. Interestingly, chloro(2,2′:6′,2″‐terpyridine)platinum acted as a strong activator of CtNDH2, increasing relative activity to 186.05%.

## 4 DISCUSSION

### 4.1 Biological rationale for targeting trypanothione reductase in leishmania

The identification of LiTR as a key therapeutic target for *Leishmania* infections has prompted extensive research efforts to develop selective inhibitors (Battista et al., 2020; Exertier et al., 2024). Advances in NGS technology and Protista genome sequencing initiatives (Cacho et al., 2014; De Grassi et al., 2010; Perles et al., 2025; Reis‐Cunha & Jeffares, 2024), have revealed that most trypanosomatids of the *Trypanosoma* and *Leishmania* genera possess quite complex iron and ROS metabolic networks regulated by enzymes, such as superoxide dismutase (SOD), heme‐containing ascorbate peroxidase (APX) and TR (Bringaud et al., 2006; Colasante et al., 2009). Interestingly, these dixenous organisms lack catalase. This is thought to be a consequence of hydrogen peroxide acting as a signaling molecule for the trypanosomatids, controlling the switch between the promastigote and amastigote phases of their life cycles upon infection of a vertebrate host. For this reason, the reactive oxygen species (ROS) equilibrium in dixenous trypanosomatids, including pathogens like *L. infantum*, is of high relevance for its growth, and its dysregulation might be more deleterious for the flagellate than for its host, which possesses dedicated enzymatic systems to regulate intracellular hydrogen peroxide levels to control the concentration of hydrogen peroxide within the cell (Kraeva et al., 2017; Mittra & Andrews, 2013), above all in case of resistance to traditional remedies (i.e., allopurinol and antimonials; Paradies et al., 2010; Saridomichelakis et al., 2025). For these reasons, LiTR appears as a suitable target for the development of antileishmanial agents. However, the structural similarities between LiTR and other flavoproteins raise concerns regarding off‐target interactions that may cause adverse effects in mammalian hosts (Dipol et al., 2025; Pierri & Pierri, 2026; Trisolini et al., 2019). Our study aimed to address this issue by computationally assessing the selectivity of LiTR inhibitors against structurally related enzymes starting from those naturally present in *Leishmania*, and by establishing a computational pipeline for future investigations into cross‐reactivity with other structurally related mammalian FAD/NAD(P)H‐dependent enzymes, including apoptosis inducing factor (AIF), glutathione reductase (GSR), thioredoxin reductase (TrxR) and dihydrolipoamide dehydrogenase (DLD) (Dipol et al., 2025; Pierri & Pierri, 2026; Trisolini et al., 2019).

### 4.2 Structural determinants of selectivity among FAD/NAD(P)H‐dependent dehydrogenases in *L. infantum*

Remarkably, 11 FAD/NADPH dependent dehydrogenase sequences from *L. infantum* were highlighted through reciprocal blastp analyses sharing structural homology with several flavoproteins, including a type II NADH dehydrogenase‐like protein (XP_001469921.1), a dihydrolipoamide dehydrogenase‐like enzyme (XP 001468025.1), and dienoyl‐CoA reductase (XP_001468165.1).

While these proteins perform distinct metabolic roles, their overlapping cofactor‐binding regions suggest that certain LiTR inhibitors might exhibit undesired binding to these enzymes, leading to cross‐reactivity and interactions with alternative targets. Our docking simulations revealed that several TR inhibitors displayed varying degrees of cross‐reactivity, emphasizing the importance of rational drug design to optimize selectivity.

In selected cases, docking analyses suggested that proper ligand accommodation required the dimeric assembly of the protein for ligands to properly bind the protein in their respective pocket, as was the case with RDS (sites 1 and 2), JV0 (site 2), or nitrofurazone (GCG binding region at the monomer‐monomer interface).

To preliminarily validate the docking predictions and investigate ligand target selectivity within structurally related flavoproteins, recombinant LiTR and CtNDH2 were selected for biochemical characterization and enzymatic assays. Although the *L. infantum* NDH2 homolog (XP_001469921.1) investigated through docking analyses has not yet been experimentally characterized structurally or kinetically, fold‐recognition analyses identified type II NADH dehydrogenases from *C. thermarum* and yeast among the closest structurally characterized homologs. In particular, the compared NDH2 proteins shared sequence identities exceeding 30%, while comparative structural analyses revealed a strong conservation of the FAD‐binding region and a partial conservation of the NAD‐binding region, especially in comparison with the yeast NDH2 structure 4g6h (Figure 3). For these reasons, the structurally and biochemically characterized CtNDH2 system was employed as an experimentally tractable flavoprotein model for preliminary evaluation of ligand‐dependent modulation within this enzyme family. Among the experimentally validated compounds, auranofin emerged as the most potent LiTR inhibitor, followed by chloro(2,2′:6′,2″‐terpyridine)platinum(II) and, to a lesser extent, nitrofurazone. Auranofin is an approved disease‐modifying antirheumatic drug (DMARD), originally developed for the treatment of rheumatoid arthritis, and has recently attracted attention for its antimicrobial, antiparasitic, and anticancer properties. Its established clinical use and well‐characterized pharmacological profile make it particularly attractive within a drug‐repurposing framework targeting parasite redox metabolism.

Nitrofural (nitrofurazone), previously reported to inhibit bacterial glutathione reductase, also demonstrated measurable inhibitory activity against LiTR in our assays, consistent with the conserved redox‐active architecture shared among flavin‐dependent disulfide reductases.

Notably, chloro(2,2′:6′,2″‐terpyridine)platinum(II), a terpyridine‐derived platinum complex, produced strong LiTR inhibition while simultaneously stimulating CtNDH2 activity, revealing an enzyme‐dependent dual behavior that warrants further mechanistic investigation.

It did, however, predict a high affinity for CtNDH2: the same strong LiTR inhibitor, chloro(2,2′:6′,2″‐terpyridine)platinum, which docked only weakly on LiTR, was a strong activator of CtNDH2 and coherently docked more efficiently on the latter. This observation underscores how subtle sequence differences may translate into distinct functional outcomes, reinforcing the need for combined structural and biochemical characterization when evaluating ligand selectivity.

### 4.3 Drug‐repurposing opportunities in antileishmanial therapy

From a drug‐repurposing perspective, auranofin and nitrofural appear particularly promising for targeting *Leishmania* redox metabolism (Kannigadu et al., 2022; Yıldırım et al., 2023), either as single agents or in combination with established antileishmanial therapies such as antimonials, amphotericin B, miltefosine, or allopurinol. Combination therapy may be particularly advantageous in cases of drug‐resistant leishmaniasis (Carrasco‐Martin et al., 2026; Krämer et al., 2025; Schäfer et al., 2024). In particular, both amphotericin B and miltefosine are known to induce oxidative stress and membrane‐associated damage in Leishmania parasites, and the additional inhibition of LiTR could impair the trypanothione‐dependent antioxidant buffering system, consequently increasing parasite susceptibility to oxidative damage. Remarkably, the previous clinical use of the three drugs may facilitate translational development, provided that selectivity toward parasite enzymes over host homologs is carefully validated. Compared with auranofin, nitrofurazone displayed weaker inhibitory effects toward LiTR in the present study. Accordingly, this compound may be more appropriately considered as a preliminary structural scaffold for future TR‐oriented ligand optimization rather than as a direct repurposing candidate. In addition, the antileishmanial activity historically associated with nitrofurazone has been mainly attributed to nitroreductase‐dependent bioactivation mechanisms rather than to direct LiTR inhibition.

Interestingly, the terpyridine‐derived platinum complex chloro(2,2′:6′,2″‐terpyridine)platinum(II) displayed a strong inhibitory effect on LiTR comparable to that observed for auranofin in the enzymatic assays. This behavior is supported by the presence of a stable and structurally coherent re‐docked pose within the LiTR binding region, suggesting that this compound, or structurally related TPT derivatives, deserves further evaluation as potential antileishmanial scaffolds. At the same time, a different behavior was observed for CtNDH2 (after superimposition operations), where the same compound stimulated enzymatic activity. Notably, although the refRMS obtained for chloro(2,2′:6′,2″‐terpyridine)platinum(II) within CtNDH2 was high, the predicted Ki remained in the micromolar range, suggesting that the ligand may interact with a nearby or partially overlapping region within the same structural area rather than occupying exactly the same binding pose observed in LiTR.

Such behavior is consistent with the high overall structural conservation that characterizes FAD/NAD(P)H‐dependent dehydrogenases. While the global architecture of these flavoproteins remains remarkably similar, local variations in amino acid composition within otherwise homologous binding regions may significantly influence ligand orientation and functional outcome. In this context, even subtle residue substitutions in structurally equivalent pockets may shift ligand accommodation toward adjacent micro‐regions of the binding site, potentially resulting in distinct enzymatic responses such as inhibition, activation, or neutral modulation.

### 4.4 Prioritizing cross‐reactivity testing based on docking reliability and predicted affinity

A comparative inspection of Tables 7, 8, 9, 10, 11, 12 suggests that the combination of predicted binding energy and pose reliability (refRMS) can be exploited as a rational prioritization criterion for experimental cross‐reactivity studies within the FAD/NAD(P)H‐dependent dehydrogenase family. In particular, TR inhibitors displaying negative binding energies together with refRMS values below the reliability threshold of 2.65 Å across structurally related flavoproteins consistently produced docking poses compatible with conserved cofactor‐binding or substrate‐recognition regions. Notably, experimental assays on recombinant LiTR and CtNDH2 demonstrated that some of these compounds are indeed capable of targeting similarly positioned binding regions in distinct enzymes, resulting either in comparable inhibitory effects or, in certain cases, in opposite functional modulation, as observed for chloro(2,2′:6′,2″‐terpyridine)platinum(II), which inhibits LiTR while stimulating CtNDH2. This convergence between computational predictions and in vitro observations supports the notion that negative binding energies combined with structurally coherent docking solutions (refRMS <2.65 Å) should be considered early indicators of potential cross‐reactivity. Accordingly, TR inhibitors fulfilling these criteria deserve to be prioritized for in vitro evaluation on additional FAD/NAD(P)H‐dependent dehydrogenases before testing compounds with weaker or geometrically inconsistent docking profiles, particularly when the aim is to identify novel modulators, either inhibitors or activators, within this structurally conserved enzymatic family. The comparative analysis summarized in Table 10 further supports the utility of the proposed workflow for identifying preliminary selectivity and cross‐reactivity trends within structurally related flavoproteins. In particular, compounds such as JV0, H6H, MWT, and WPF displayed docking profiles generally favoring LiTR over the investigated human homologs, whereas TS8 and BVN exhibited partially overlapping affinity trends suggestive of a broader flavoprotein interaction potential. Although these observations require dedicated biochemical validation, they highlight the possibility of prioritizing ligands according to their predicted selectivity or cross‐reactivity profiles during the early stages of flavoprotein‐oriented drug discovery.

Nevertheless, some methodological limitations associated with the present docking strategy should be considered. The implemented workflow was conceived as a rapid comparative screening approach aimed at evaluating relative ligand selectivity trends across a broad set of structurally related FAD/NAD(P)H‐dependent dehydrogenases. Accordingly, docking calculations were performed using rigid receptor structures while maintaining ligand flexibility whenever computationally feasible, an approach particularly suited for comparative large‐scale analyses across multiple homologous proteins and binding regions.

In this context, the adopted docking protocol did not explicitly account for large‐scale protein flexibility or solvent‐mediated interactions during ligand binding. For these reasons, the adopted refRMS threshold should be interpreted as an empirical indicator of docking pose consistency rather than as an experimentally validated cutoff. Accordingly, docking‐derived affinities and inhibition constants were interpreted comparatively across structurally related systems and integrated with structural superimposition analyses and biochemical validation data rather than being considered absolute predictors of ligand binding.

An additional methodological consideration on the present study concerns the investigated inhibitor library, primarily composed of crystallographically characterized compounds or ligands associated with structurally resolved binding regions. This choice was intentionally adopted to enable comparative re‐docking analyses and facilitate identification of conserved ligand‐binding regions across structurally related flavoproteins with improved structural interpretability. Nevertheless, the analyzed compound set remains relatively limited in terms of structural diversity. Future extensions of the proposed workflow should include broader diversity‐based virtual screening campaigns, pharmacophore‐oriented analyses, and more advanced computational approaches integrating molecular dynamics simulations, explicit solvent models, and free‐energy‐based calculations to further refine the assessment of ligand selectivity and support dedicated validation studies on selected protein–ligand complexes identified through the present strategy.

### 4.5 Broader translational implications beyond leishmania

Extending beyond the parasite context, the risk of unintended host enzyme inhibition remains a critical consideration in drug development. In this regard, the provided pipeline can be employed to check possible cross‐reactivity of the investigated inhibitors with mammalian homologs, including AIF, GSR, TrxR, and DLD, all of which share structural and functional similarities with LiTR and with the other FAD/NAD(P)H‐dependent dehydrogenases highlighted in *L. infantum* (Trisolini et al., 2019).

**FIGURE 9:**
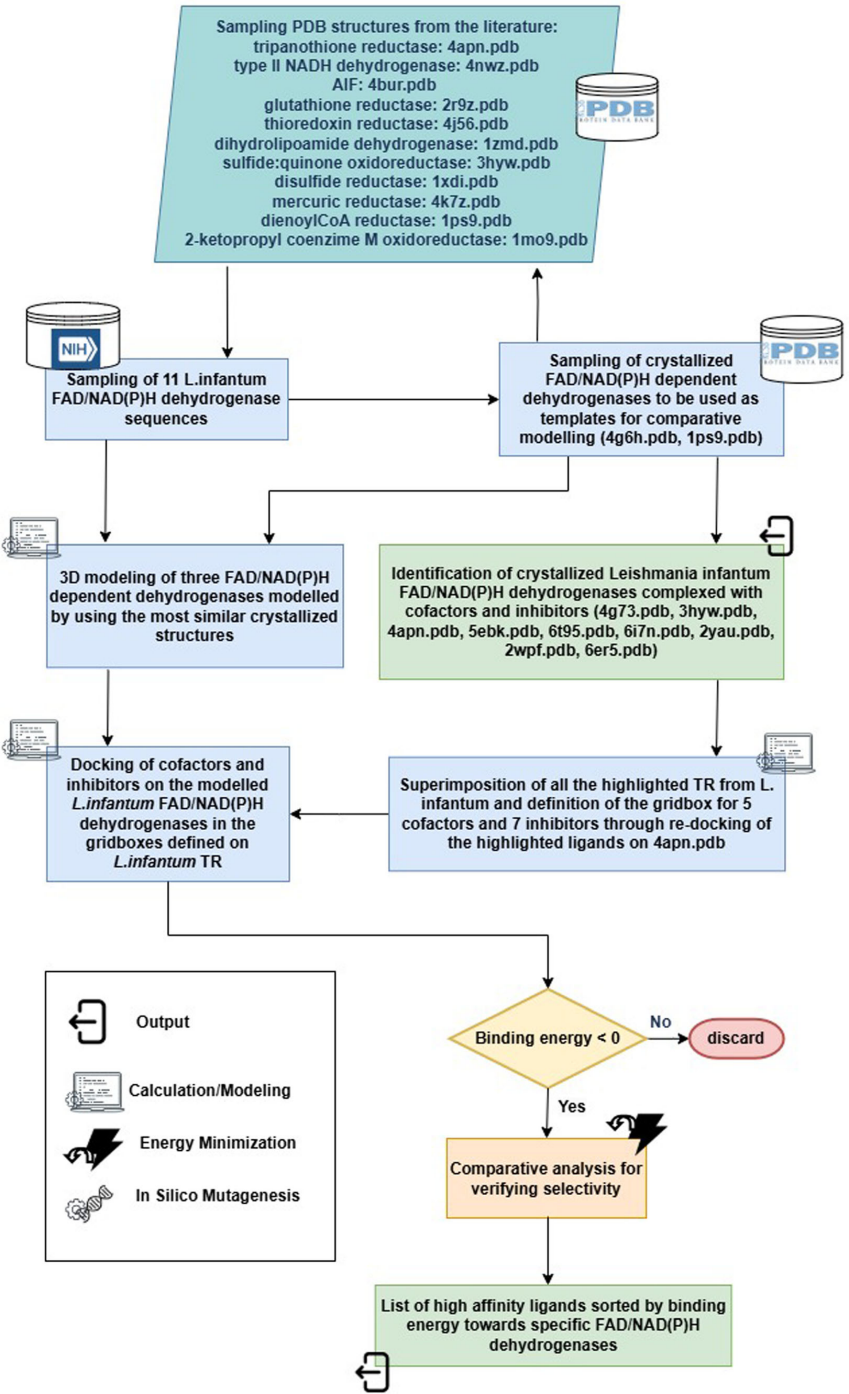
Overview of the computational workflow used for the identification and selectivity assessment of ligands targeting trypanothione reductase within the broader family of FAD/NAD(P)H‐dependent dehydrogenases. Crystallized FAD/NAD(P)H‐dependent dehydrogenases were first collected from the Protein Data Bank (PDB) based on literature mining. In parallel, flavoprotein sequences from *Leishmania infantum* were identified and clustered. Selected *L. infantum* enzymes were modeled in three dimensions using the most appropriate crystallized homologs as templates. Known cofactors and inhibitors were extracted from available crystallographic complexes and re‐docked on *L. infantum* trypanothione reductase to define binding regions and grid boxes. Docking simulations of cofactors and inhibitors were subsequently performed on both crystal structures and homology models. Ligands displaying favorable binding energies were retained for comparative analysis across parasite and human FAD/NAD(P)H‐dependent dehydrogenases, enabling the evaluation of binding trends and selectivity profiles.

The proposed workflow (Figure 9) provides a structural strategy to evaluate and refine ligand selectivity across parasite and host homologs. At the same time, in the context of the drug‐repurposing approaches, the highlighted FAD/NAD(P)H‐dependent dehydrogenase inhibitors can be used as scaffolds for designing new ligands with an improved affinity toward human FAD/NAD(P)H dehydrogenases, including the above mentioned AIF, GSR, TrxR, and DLD often dysregulated in cancer or rare diseases (Bano & Prehn, 2018; Bjørklund et al., 2021; Carrozzo et al., 2014; Ferraris et al., 2020; Ghezzi et al., 2010; Herrmann & Riemer, 2021; Kalinina, 2024; Kamerbeek et al., 2007; Leone & Powell, 2020; Lopert et al., 2012; Misevičien et al., 2011; Modjtahedi et al., 2010; Monteiro et al., 2017; Nguyen & Pandey, 2019; Palmieri et al., 2023; Patwardhan et al., 2022; Urbano et al., 2005; Xu et al., 2024; Yumnam et al., 2021; Zhang et al., 2023). Indeed, beyond antiparasitic applications, the differential modulation of FAD/NAD(P)H‐dependent dehydrogenases observed in this study may provide insights for broader pharmacological strategies. Selective inhibition of bacterial NDH2 could support antibiotic development, whereas controlled modulation of human flavoproteins involved in redox homeostasis may be relevant for pathological conditions characterized by altered NAD+/NADH or FAD/FADH2 balance, including certain cancers and rare metabolic disorders (Dipol et al., 2025; Trisolini et al., 2019). Although these implications remain to be experimentally validated, they illustrate the broader translational potential of the structural framework presented here (Heikal et al., 2014; Manap et al., 2026; Trisolini et al., 2019).

More generally, the present findings highlight the importance of extending selectivity analyses across structurally related flavoproteins before advancing candidate molecules toward therapeutic development. In particular, the present workflow could be further exploited through additional in silico analyses aimed at investigating structurally similar and spatially equivalent binding regions across homologous enzymes in both parasite and host systems. Systematic exploration of these locally conserved pockets may help identify structural determinants responsible for enzyme‐specific responses and guide the rational optimization of ligand selectivity.

In a broader pharmacological perspective, the possibility that certain molecules may inhibit parasite flavoproteins while preserving—or even stimulating—selected host redox enzymes raises intriguing therapeutic opportunities. Such differential modulation could, in principle, contribute to pathogen eradication while maintaining host redox homeostasis and potentially minimizing detrimental effects on beneficial members of the human microbiome, also in the context of rescue‐pharmacology approaches (Pierri & Pierri, 2026). Although these hypotheses require further experimental validation, they illustrate how the combined computational–biochemical strategy proposed here may facilitate the identification of flavoprotein modulators with favorable selectivity profiles across complex biological systems.

## 5 CONCLUSIVE REMARKS

In conclusion, this study defines a structurally informed and experimentally supported framework for assessing the selectivity of trypanothione reductase (TR) inhibitors within the broader family of FAD/NAD(P)H‐dependent dehydrogenases. By integrating comparative docking analyses with biochemical validation on recombinant LiTR and CtNDH2, we show that structurally related flavoproteins can accommodate shared ligands within conserved binding regions, resulting in either inhibitory or enzyme‐specific modulatory effects.

Importantly, the combined evaluation of predicted binding energies and docking reliability (refRMS) emerges as a practical prioritization criterion for identifying compounds that merit further in vitro cross‐reactivity testing. TR inhibitors displaying negative binding energies together with structurally coherent docking poses (refRMS <2.65 Å) across related enzymes should be considered early candidates for expanded enzymatic profiling, both to anticipate potential off‐target interactions and to uncover multi‐target modulation within this redox‐active enzyme family.

The proposed computational and biochemical workflow (Figure 9) therefore represents not only a tool to refine antileishmanial drug discovery strategies but also a transferable approach for investigating ligand selectivity across structurally conserved flavoproteins. The experimental validation supporting auranofin, nitrofural, and terpyridine‐derived platinum compounds as direct enzymatic modulators strengthens the translational perspective of this study while emphasizing the necessity of systematic evaluation against host homologs.

Given the evolutionary conservation of FAD/NAD(P)H‐dependent dehydrogenases, these findings may extend beyond the parasite system and provide a structural rationale for the modulation of related enzymes implicated in infectious, metabolic, or redox‐associated human diseases. Further investigations integrating molecular dynamics simulations together with expanded enzymatic and cellular validation will be essential to more precisely define the therapeutic window and translational applicability of this strategy.

## AUTHOR CONTRIBUTIONS

**Serena Spadone:** Methodology; software; formal analysis; writing – original draft; writing – review and editing. **Stefania Varani:** Conceptualization; writing – original draft; writing – review and editing; supervision. **Margherita Ortalli:** Methodology; formal analysis; investigation. **Sara Morselli:** Methodology; formal analysis; investigation. **Giulia Chiara Maria Perrone:** Methodology; software; formal analysis; writing – original draft; writing – review and editing. **Domenico Otranto:** Conceptualization; writing – original draft; funding acquisition; writing – review and editing; supervision. **Jairo Alfonso Mendoza‐Roldan:** Methodology; formal analysis; investigation. **Federica Belluti:** Conceptualization; writing – original draft; writing – review and editing; supervision. **Lorenzo Guerra:** Supervision; writing – review and editing. **Ciro Leonardo Pierri:** Conceptualization; funding acquisition; writing – original draft; writing – review and editing; supervision; software; formal analysis. **Maria Noemi Sgobba:** Methodology; formal analysis; investigation. **Mariateresa Volpicella:** Conceptualization; writing – original draft; writing – review and editing; supervision. **Sabino Todisco:** Methodology; formal analysis; investigation. **Valeria Scaglione:** Methodology; formal analysis; investigation. **Anna Lucia Francavilla:** Methodology; formal analysis; investigation. **Anna De Grassi:** Conceptualization; writing – original draft; writing – review and editing; supervision.

## CONFLICT OF INTEREST STATEMENT

The authors declare no conflicts of interest.

## Supporting information


**Data S1:** Supporting information.

## Data Availability

The data that support the findings of this study are available from the corresponding author upon reasonable request. Data will be made available on request.
