# Supplementary material for: Structural basis of ligand selectivity in FAD/NAD(P)H‐dependent dehydrogenases: insights from trypanothione reductase and type II NADH dehydrogenase
Source: Protein Sci. 2026 Jun 20;35(7):e70664. doi: 10.1002/pro.70664 (PMC13282843; doi:10.1002/pro.70664)

Lysis and Purification LiTR

SDS-PAGE lysis sonication BL21(DE3) + LiTR

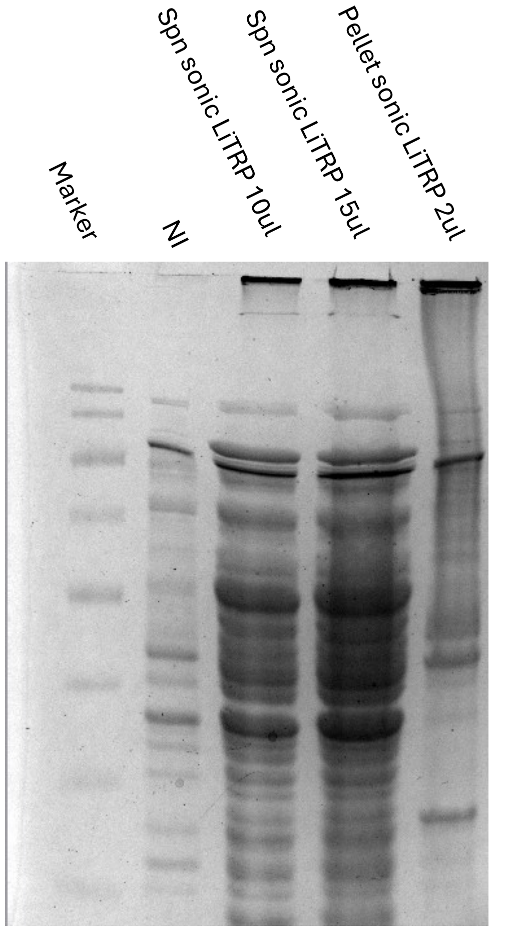

SDS-PAGE purification LiTR through His-trap from spn sonication

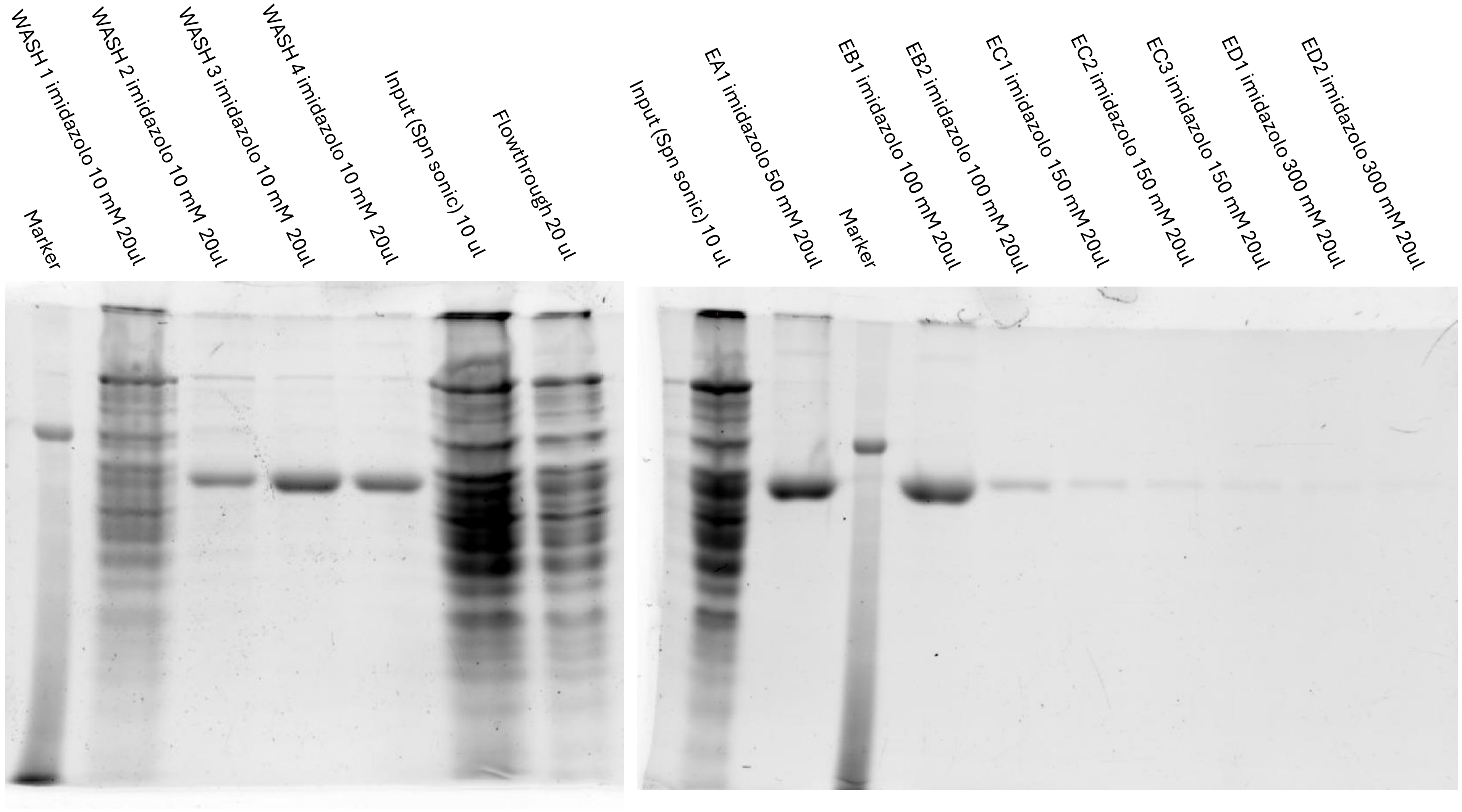

Lisi e purificazione CtNDH2

SDS-PAGE lisato sonicatore C41(DE3) + CtNDH2

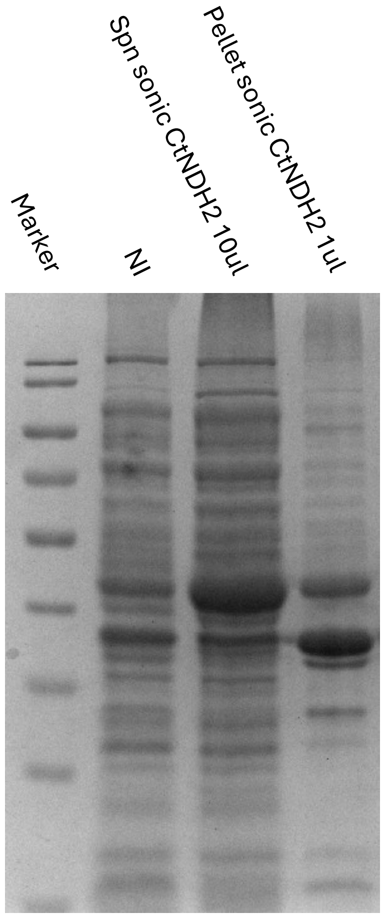

SDS-PAGE purificazione CtNDH2 tramite His-trap da spn sonicatore

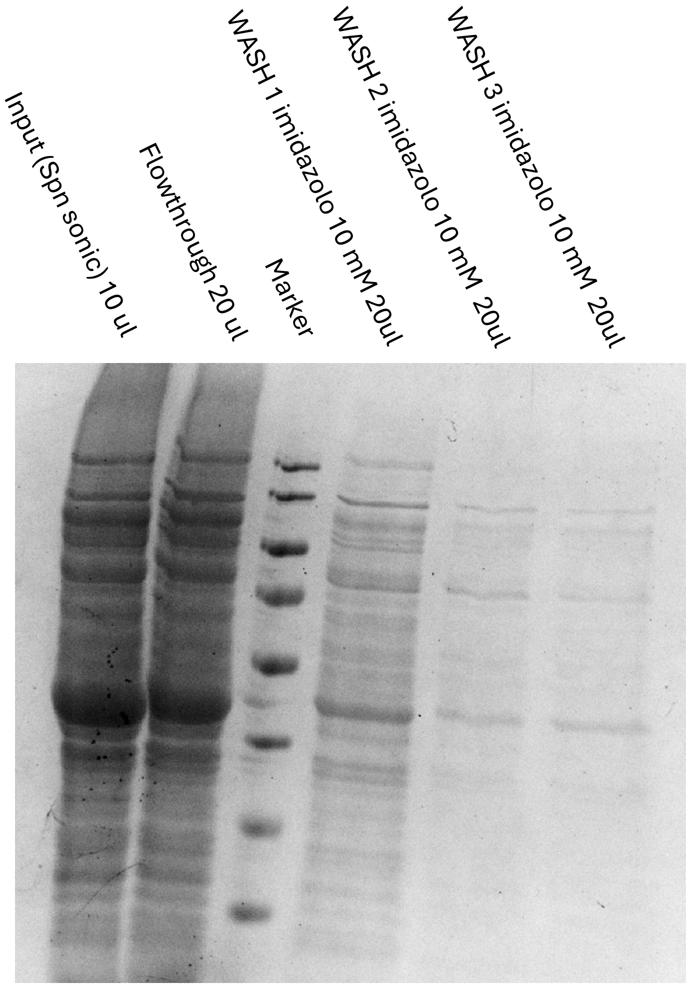

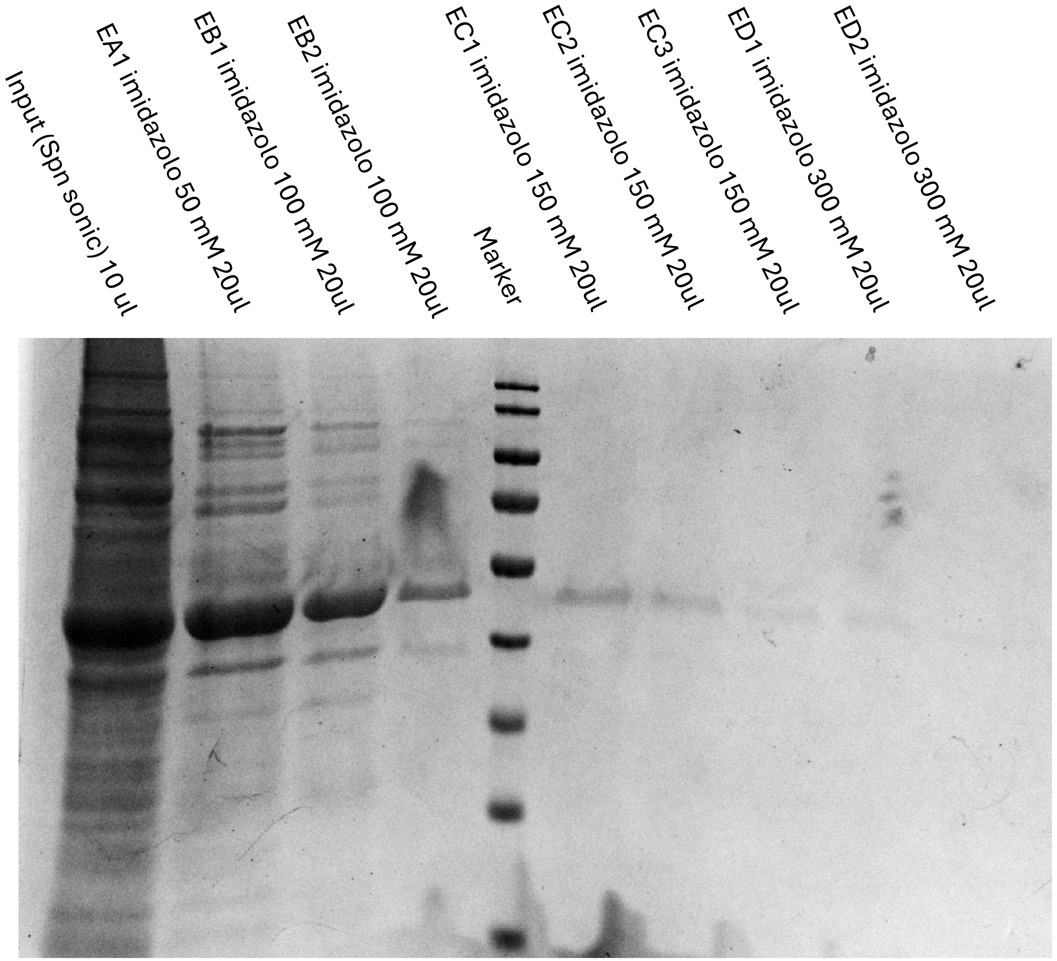

Imagine for the paper

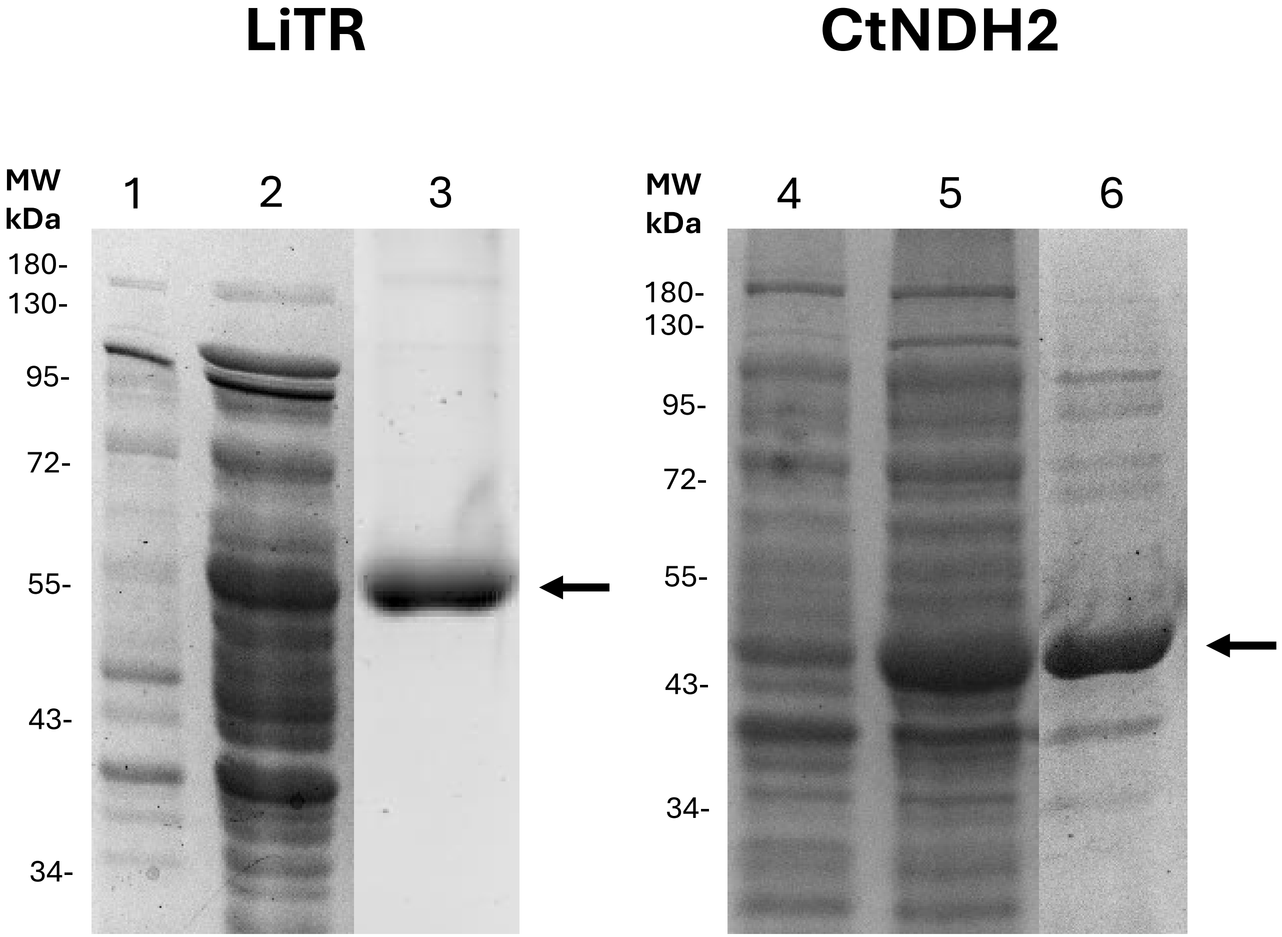

LiTR

BSA 1ug/ul
BSA 2ug/ul
BSA 3ug/ul
BSA 4ug/ul
BSA 5ug/ul
LiTR post AMICON 1:10 5ul
LiTR post AMICON 1:10 10ul
LiTR post AMICON 1ul

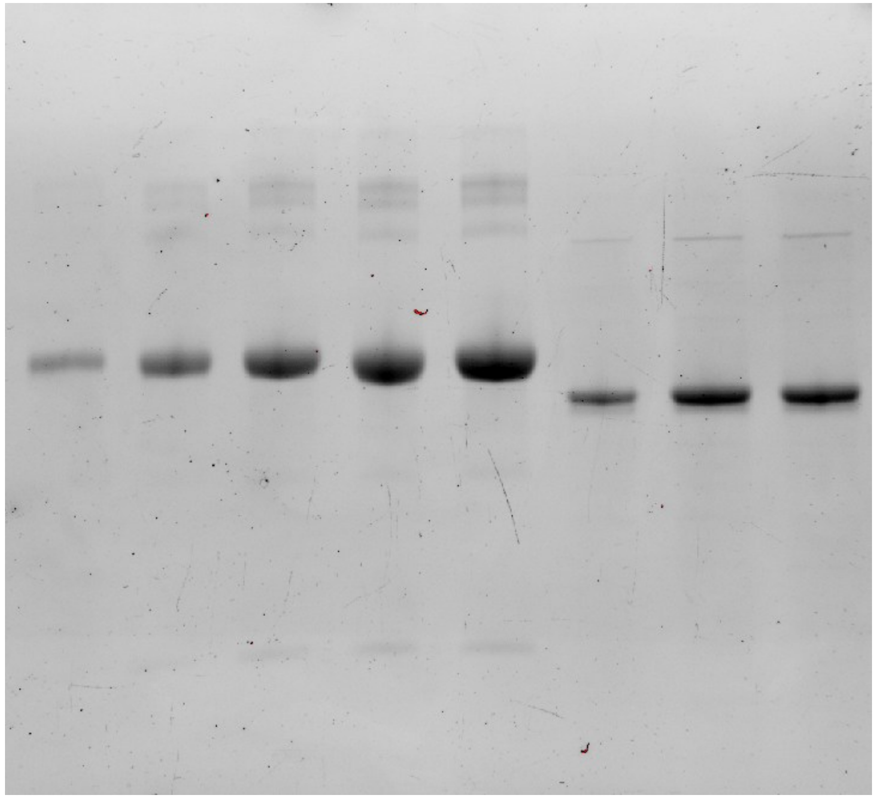

CtNDH2

BSA 1ug/ul
BSA 2ug/ul
BSA 3ug/ul
BSA 4ug/ul
BSA 5ug/ul
CtNDH2 post AMICON 2ul
CtNDH2 post AMICON 5ul
CtNDH2 post AMICON 10ul

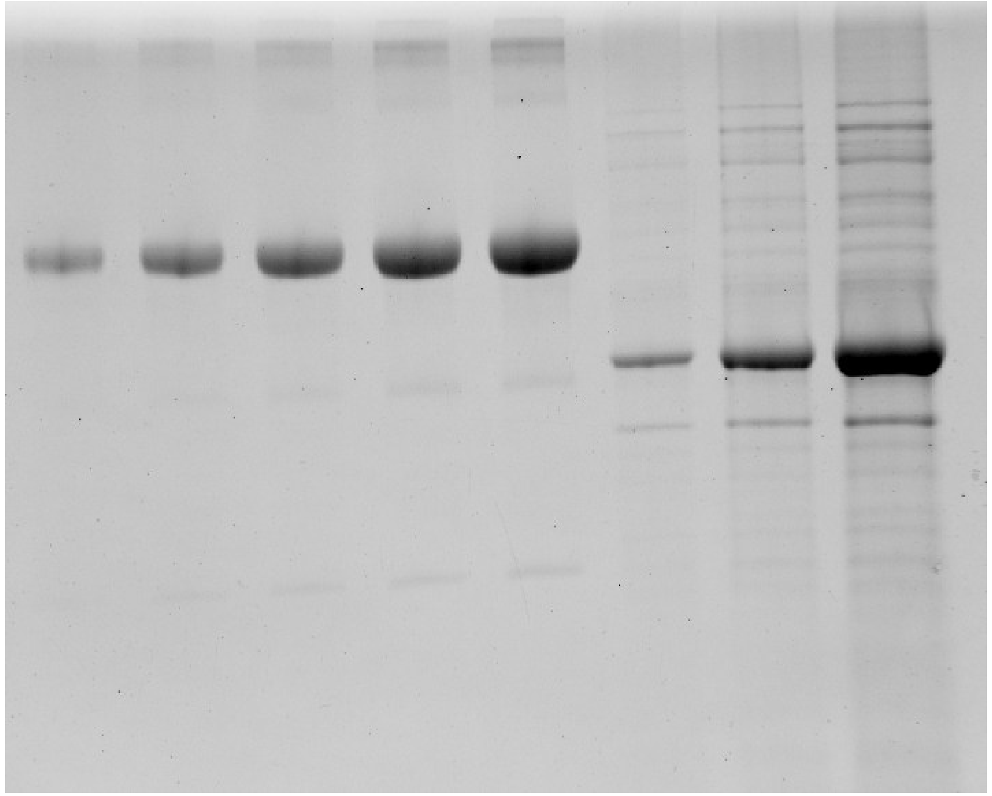

Supplement: Supplementary file 1 — Data S1: Supporting information. [file PRO-35-e70664-s001.pdf]
